# Operationalizing the Exposome Using Passive Silicone Samplers

**DOI:** 10.1007/s40726-021-00211-6

**Published:** 2022-01-04

**Authors:** Zoe Coates Fuentes, Yuri Levin Schwartz, Anna R. Robuck, Douglas I. Walker

**Affiliations:** Department of Environmental Medicine and Public Health, Icahn School of Medicine at Mount Sinai, 1428 Madison Ave, New York, NY USA

**Keywords:** Exposome, Precision medicine, Silicone wristband samplers, Exposure assessment, High-resolution mass spectrometry

## Abstract

The exposome, which is defined as the cumulative effect of environmental exposures and corresponding biological responses, aims to provide a comprehensive measure for evaluating non-genetic causes of disease. Operationalization of the exposome for environmental health and precision medicine has been limited by the lack of a universal approach for characterizing complex exposures, particularly as they vary temporally and geographically. To overcome these challenges, passive sampling devices (PSDs) provide a key measurement strategy for deep exposome phenotyping, which aims to provide comprehensive chemical assessment using untargeted high-resolution mass spectrometry for exposome-wide association studies. To highlight the advantages of silicone PSDs, we review their use in population studies and evaluate the broad range of applications and chemical classes characterized using these samplers. We assess key aspects of incorporating PSDs within observational studies, including the need to preclean samplers prior to use to remove impurities that interfere with compound detection, analytical considerations, and cost. We close with strategies on how to incorporate measures of the external exposome using PSDs, and their advantages for reducing variability in exposure measures and providing a more thorough accounting of the exposome. Continued development and application of silicone PSDs will facilitate greater understanding of how environmental exposures drive disease risk, while providing a feasible strategy for incorporating untargeted, high-resolution characterization of the external exposome in human studies.

## The Exposome

The past 10 years have seen rapid growth in exposomic studies, providing improved understanding of how environment and other non-genetic factors contribute to disease risk. First defined by Christopher Wild in 2005, the exposome was envisioned as a complement to the genome that aims to define an individuals’ history of exposure and relationship with disease risk, including the influence of lifestyle, diet, and the environment [1]. This definition was expanded by Rappaport and Smith to separately consider exposures external (bottom-up) and internal (top-down) to the host [2, 3].

Recognizing the importance of biological response, the exposome concept has since been revised by Miller and Jones to: *The cumulative measure of environmental influences and corresponding biological responses throughout the lifespan, including exposures from environment, diet, behavior, and endogenous processes* [4]. Within this conceptualization, exposures include the continuum from external stressors, processes internal to the body, socioeconomic influences, and psychological factors. This characterization of the exposome as a combination of exposure and biological response is referred to as functional exposomics and emphasizes multi-omic and systems biology strategies to systematically study the interaction of environment, host response, and disease [5, 6].

By providing a key measure of non-genetic disease risk factors, the exposome has the potential to broaden understanding of how environmental stressors contribute to disease [6]. However, unlike genetic sequencing, measuring the cumulative effect of exposure over the lifespan comes with significant complications. Measurements of chemical exposure are compounded by the estimated millions of exposures that vary temporally over a lifetime, including environmental pollutants, chemicals in consumer-facing goods, biologics, and pharmaceuticals [7]. The relationship between many of these exposures and health effects is unknown, and there is a need to perform discovery studies that enable systematic characterization of the exposome and how it relates to health outcomes. These exposome-wide association studies require robust sampling strategies that can be incorporated into population studies and enable detection of a wide range of exposures. In this review, we highlight the advantages of considering measurement of the untargeted external exposome for new insight into the relationship between environmental exposures and disease. We discuss the use of silicone passive sampling devices (PSDs), which show considerable promise for untargeted measurement of external exposures within an exposome-wide association study framework. Analytical considerations, including untargeted analysis using high-resolution mass spectrometry and strategies for exposome data science, are discussed. Lastly, we provide a framework for using untargeted external exposure monitoring within a deep exposome phenotyping framework.

## Operationalization of the Human Exposome

The exposome is unique in ‘omic sciences because it represents an integrated measure across multiple compartments characterizing how non-genetic factors external and internal to the host influence disease risk. As a result, the exposome in its totality requires attention to impacts from internal, external, and psychosocial factors, many of which require separate approaches and study designs to measure. The internal exposome combines measurement of biological activity with internal dose biomarkers that can include levels of the parent chemical, transformation products, and adducts of reactive compounds. The external exposome includes environmental exposures to toxic chemicals, pollution, and radiation, while also incorporating behavioral variables like diet, exercise, and drug use. Non-specific exposures, such as social and psychological stressors, are the third component of the exposome. While these three compartments tend to be measured and considered separately, they are dependent and interrelated. All exogenous biomarkers (internal exposome) originate from exposures that occurred outside the host (external exposome), and many of these exposures can be both potentiated and varied depending on non-specific factors, such as stress and lack of sleep (psychosocial exposome) [8].

### Measuring the Internal Exposome

Most efforts to date have focused on the internal exposome due to the availability of biological specimens collected and stored from well-established population studies. These include approaches that aim to identify chemical biomarkers to estimate exposure burden and its relationship to adverse health outcomes, as well as ‘omic approaches that define specific phenotypes of exposure and disease. The most promising approaches for comprehensive measures of the internal exposome include untargeted high-resolution metabolomics, which detects low molecular weight compounds within a biological sample [9–11]. While initially developed to characterize disease-related changes in endogenous metabolites, methods that use high-resolution mass spectrometry (HRMS) show sensitivity and dynamic range to detect low-level chemical exposures and drugs, in addition to endogenous metabolites from critical pathways [9, 12, 13]. Continued advancement in HRMS instrumentation and computational approaches for data extraction has resulted in their widespread adoption for exposome research [14–16]. New applications show the strengths of HRMS to understand chemical phenotypes of exposure for environmental stressors, exposures during pregnancy and other life stages, occupational exposures, and environment-disease relationships [17–25]. When combined with additional ‘omic measures, HRMS provides a systems biology approach to link exposure to internal dose, biological response, and disease [6]. Within this framework, internal dose is assessed by screening for the presence of metabolites that arise from exogenous chemicals, while biological response to exposure is determined by identifying alteration in endogenous processes (e.g., gene, protein, and metabolite expression). Biological alterations associated with exposure or disease can be considered separately using a “meet-in-the-middle” approach, and overlapping associations reinforce a causal relationship between exposure and disease, providing insight into underlying disease mechanisms [26–28]. However, interpretation of these results can be challenging due to exposure timing, varied or unknown biological half-lives of exposure biomarkers, and complex exposure–response effects that occur in distal tissues.

### Measuring the External Exposome

External exposome monitoring provides a standalone, but complementary, measure of environmental stressors [29]. Unlike measurements for the internal exposome, which tend to be precise to the individual, precision for the external exposome varies depending on measurement strategy. When estimating inhalation and location-driven exposures for populations over large geographical areas, geospatial/remote sensing and regional stationary sampling approaches are often used [30–32]. Air pollution is often assessed with satellite-based surface-point differentiation, and remote sensing methods have also been used to assess distance to green and blue space, temperature, and light pollution [33]. Chemical exposures can be estimated using distance from known pollution sources, such as location relative to contaminated sites, or surface and groundwater pollution [34]. Stationary samplers that incorporate sensors can provide highly accurate measurements at a single location over time, while others that use absorbent sampling material to collection pollutants provide an integrated measure over the observation period. These point-measurements from stationary samplers are then often extrapolated to estimate regional concentrations. Depending on the age of the samplers or satellites, these approaches provide temporally dense measures to estimate exposure histories for large populations, a key advantage when studying how past exposures influence current health outcomes [35–42]. However, many of the techniques used to estimate exposures are limited and lack the precision to assess microenvironment changes. The use of mobile sampling devices, including automobiles or drones that include adjustment for time and activity patterns using smart phones, has improved accuracy; however, these approaches still cannot account for high variability due to activity and changes in microenvironment exposure levels.

Individual exposome monitoring focuses on characterizing interactions between a person and exposure sources. As a result, multiple strategies are possible, including detection of chemical exposures in food or water and characterization of the indoor and outdoor microenvironments [43]. To measure inhalation exposures, mobile samplers are often worn by study participants or can be placed throughout different microenvironments to improve measurement resolution. These samplers can be active, which combines a pump with samplers to quantitatively measure exposure, as well as PSDs, which collect time-integrated concentrations of chemicals through passive diffusion into the sampler matrix. Though various designs for active samplers have been developed, key limitations include the need for an external battery to power sampling pumps, frequent calibration to verify air flow, and expensive equipment. Thus, active samplers can be difficult to operate and uncomfortable to carry, especially for children [44–47]. Passive samplers, which rely on less invasive technologies such as adsorbent strips and wearables like silicone wristbands, pouches, and badges, provide an alternative strategy to screen for both known and unknown exposures in large populations. PSDs validated for exposures with known uptake rates, such as benzene or trichloroethylene, have been widely used in occupational monitoring studies for industrial chemicals [23, 48–54]. However, for most PSDs, ongoing research is focused on better understanding the mechanisms for chemical equilibrium with sampler matrices, which can vary by chemical molecular weight, media pore size, and silicone/air partitioning coefficients [55]. Determining these parameters for different PSD materials and designs is necessary for understanding biases when this approach is used for exposure monitoring.

To better incorporate the exposome into the study of human health, there is a critical need to leverage strategies that enable comprehensive characterization across different exposome compartments. To achieve the power necessary to identify how low-level exposures and associated mixture effects contribute to disease outcomes, it is necessary to use approaches that allow low-cost sampling options and can be deployed in large populations. Current studies show how biospecimens, including blood, urine, and saliva, combined with untargeted assays, provide a solution for internal exposome characterization [7, 56–59]; however, no similar approaches are routinely available for the external exposome. In the following sections, we review the use of innovative silicone PSDs that show considerable promise as sampling devices to screen the external exposome.

## Passive Sampling Devices for External Exposome Profiling

PSDs are non-invasive, easy to distribute, and can overcome many of the limitations that complicate interpretation of exposure biomarkers in biological samples [60]. While the configuration and material can vary, resulting in differences in uptake kinetics and exposure sampling, PSDs generally include some type of sorbent material allowing diffusion within the sorbent matrix following air or surface contact. Ideal sampling materials show linear uptake, high capacity, and reproducible sampling behavior under typical deployment conditions. When displaying these properties, PSDs have the ability to collect a time-averaged, personalized measurement of respiratory and/or dermal chemical exposures. Many of these properties are dependent on sampler material, the analyte of interest, and the sampler design, as such, PSD validation for specific analytes may be needed if strict quantitation is required. PSD configurations and placement can also be optimized to detect specific routes of exposure. For example, some are designed to only measure airborne exposures by minimizing contact with media other than air, either by encasing sampling material or by placing as a brooch over clothing [61–63]. Others, such as wristbands, show promise as an integrated measure of multiple exposure pathways [64].

Ideal PSDs for exposomic studies should have high partitioning coefficients for compounds with a wide range of physiochemical properties, be cheap to manufacture, and be provided in a form that is easy for the participant to use. While multiple strategies have been proposed, the use of commercially available silicone wristbands has shown to provide a versatile, low-cost PSD that enables screening for a broad range of chemical exposures [65–67]. As a result, use of PSDs that are primarily composed of polydimethylsiloxane and other silicone elastomers are one of the more commonly used sampling materials for PSDs. In Table 1, we summarize the human exposure studies completed to date that leveraged commercially available silicone materials as PSDs for human exposure monitoring.

**Table 1:** Summary of silicone PSD use to study environmental exposures in human populations

**PSD form factor**	**Platform used**	**Analysis approach**^1^	*N* **study participants**	**Targeted chemicals reported**	**Screened chemicals reported**	**Chemical signals from untargeted reported**^2^	**Chemical classes detected**^3^	**Length of time worn**	**Silicone conditioning**^4^	**Sample preparation method**	**Comp. measures**	**Ref**
Wristband and anklet	GC × GC-QTOF	U	2	-	-	332	VOCs	4 h and 9 h	Solvent conditioning using 3 rounds of 1:1 acetone and methanol	Thermal desorption	Questionnaire	[79]
Wristband	GC-ECD	T	35	26	-	-	Pyr, OPP	< 5 d	Heating in vacuum oven at 280–300 °C for 48 h	Extracted twice using 100 mL ethyl acetate and concentrated to 1 mL	Pesticide use records	[60]
Wristband	GC-ECD	T	10	20	-	-	OCP, OPP, pyr, phenylpyrazoles, neonicotinoids	7–14 d	NR	Extracted twice using 100 mL ethyl acetate and concentrated to 1 mL	DNA damage biomarkers	[72]
Wristband	GC-HRMS	T	88	13	-	-	OPEs	5 d	Solvent conditioning with 1:1 ethyl acetate/hexane followed by 1:1 ethyl acetate/methanol for 12 h	0.8 g WB were extracted 3 times using 1:1 hexane:dichloromethane; combined extracts were concentrated to 1 mL and loaded onto florisil column. Columns were washed with 40 mL of hexane and 40 mL of ethyl acetate. Samples were dried and reconstituted in 1 mL hexane prior to analysis	Daily surveys	[73]
Wristband	GC-HRMS	SS, U	3	-	32	163	Pesticides, FR, PCB, OPE, PAH	7 d	Solvent conditioning including 3 × 1:1 ethyl acetate/hexane and 2 × 1:1 ethyl acetate/methanol for 2.5 h each	2 g WB were extracted twice using 25 mL ethyl acetate on orbital shaker for 2 h. Extracts were loading onto SPE cartridge and washed with 6 mL AcCN. Extracts were dried and resuspended in isooctane	NR	[74]
Fresh Air sampler	GC-HRMS	U	32	-	-	275	VOCs, SVOCs, NVOCs, PAHs, PCPs, dyes, pollutants	24 h	Thermal conditioning	Thermal desorption	PSD placed on chest, wrist, and shoe placement, activity log with questionnaire, house characteristic form	[61]
Fresh Air sampler	GC-HRMS	U	84	-	-	615	PAHs, VOCs, insecticides, food flavorings, consumer products	3 d	Thermal conditioning	Thermal desorption	Outdoors passive sampling, questionnaire	[63]
Wristband	GC–MS	T	71	41	-	-	PAHs	1 d	Solvent conditioning with methanol for 10 min, followed by 3 × 1:1 ethyl acetate/hexane for 1 h each, 2 × 1:1 ethyl acetate/methanol on orbital shaker	WB were washed in methanol and extracted twice for 1 h with 30 mL ethyl acetate on an orbital shaker at 120 RPM. Dried to 2 mL using N2 evaporation	Survey	[152]
Wristband	GC–MS	T	15	18	-	-	PAHs	1 d	Solvent conditioning with methanol for 10 min, followed by 3 × 1:1 ethyl acetate/hexane for 1 h each, 2 × 1:1 ethyl acetate/methanol on orbital shaker	WB were washed in methanol and extracted twice for 1 h with 30 mL ethyl acetate on an orbital shaker at 120 RPM. Dried to 2 mL using N2 evaporation	Survey	[153]
Wristband	GC–MS	SS	255 and 20	-	199	-	PAHs	7 d	Heat conditioning in vacuum oven at 300 °C and 0.1 Torr for 12 h	WB rinsed 2 × with DI water and isopropanol. Prior to extraction, wristbands were stored in amber glass jars at − 20 °C. Deuterated analytes were added as recovery surrogates. WB extracted wristbands with 2 × 50 mL volumes of ethyl acetate. Sample extracts were reduced to 1 mL with N2. 100 µL extracts were loaded onto C18 SPE cartridges and eluted with acetonitrile. Extracts were solvent exchanged to isooctane	Survey	[154]
Wristband	GC–MS	T	40	4	-	-	OPFRs	5 d	Solvent conditioning with 1:1 ethyl acetate/hexane followed by 1:1 ethyl acetate/methanol for 12 h	WB extracted with 1:1 hexane:acetone for 12 h concentrated using N2. Extracts were filtered with a 25 mm syringe filter with a 0.2 μm PTFE membrane to remove larger particles and cleaned using Florisil SPE. Analytes were eluted with 10 mL of hexane and then 10 mL ethyl acetate. Each fraction was concentrated to 1 mL using N2 prior to analysis	Urine, hand wipes, questionnaire	[87]
Wristband	GC–MS	T	30	26	-	-	BFRs	7 d	Solvent conditioning with 1:1 ethyl acetate/hexane followed by 1:1 ethyl acetate/methanol for 12 h	WB cut into 8 equal pieces and extracted 3 × with 10 mL 1:1 hexane/acetone in a sonicating bath and concentrated to 1 mL using N2. Extracts were collected into 2 separate fractions using florisil SPE; the fraction 1 was eluted with 8 mL hexane and fraction 2 with ethyl acetate. Fraction 1 was further purified using 12 g of deactivated silica gel with 5% sulfuric acid and eluted using 1:1 hexane/dichloromethane. Fraction 1 was reduced to 1 mL using N2 prior to analysis	Serum biomarkers, questionnaire	[88]
Wristband	GC–MS	T	101	9	-	-	PAHs	7 d	Solvent conditioning with 1:1 ethyl acetate/hexane followed by 1:1 ethyl acetate/methanol for 24 h	WB extracted 2 × with 30 mL hexane/acetone for 2 h. Extracts were concentrated using N2 evaporation, purified using SPE with 40 mL DCM and reduced to 1 mL using N2	Indoor and outdoor PUF disk passive air samplers for 28 days, questionnaires	[93]
Wristband	GC–MS	T	92	20	-	-	BFRs	7 d	Solvent conditioning with 3 × 1:1 ethyl acetate/hexane followed by 2 × 1:1 ethyl acetate/methanol on orbital shaker for > 2.5 h at 30 °C	WB extracted 2 × with 100 mL of ethyl acetate. Extract was combined and reduced to 300 µL with N2. 3 mL of acetonitrile was added to each extract and loaded onto 500 mg C18 SPE cartridges (pre-rinsed with 6 mL of acetonitrile. Analytes were eluted with 9 mL of acetonitrile, reduced to 0.5 mL, and solvent-exchanged to hexane	Survey	[98]
Wristband or anklet	GC–MS	T	69	≥ 11	-	-	BDEs, OPFRs	7 d	Solvent conditioning with 3 × ethyl acetate/hexane followed by 2 × ethyl acetate/methanol after minimum of 2.5 h on an orbital shaker at 60 rpm	WB extracted 2 × with 100 mL of ethyl acetate. Extract was combined and reduced to 300 µL with N2. 3 mL of acetonitrile was added to each extract and loaded onto 500 mg C18 SPE cartridges (pre-rinsed with 6 mL of acetonitrile. Analytes were eluted with 9 mL of acetonitrile, reduced to 0.5 mL, and solvent-exchanged to hexane	Social skills improvement, system social behavior assessment	[96]
Brooch, wristband, armband	GC–MS	T	45	37	-	-	PBDEs, NHFRs, OPEs	8 h	Solvent conditioning with ethyl acetate/hexane for 3 days followed by methanol for 1 day	WB extracted in 30 mL acetonitrile using a wrist action shaker and reduced to 0.5 mL N2. Extracts were filtered through a 17 mm Teflon syringe filters with pore size of 0.2 μm	Personal active air sampler, OSHA versatile sampler, plasma and urinary biomarkers	[68]
Wristband, cut wristband pinned as a lapel, stacked wristband (to protect from sweat)	GC–MS	T, SS	22, 8	23	49	-	PAHs, PCPs, pesticides, phthalates, industrial compounds	8 h, 32–39 h, 30 d	Solvent conditioning with 3 × 1:1 ethyl acetate/hexane followed by 2 × 1:1 ethyl acetate/methanol on orbital shaker for > 2.5 h	WB rinsed 2 × with DI water and IPA. WB were extracted 2 × 100 mL of ethyl acetate 2 h each. Extracts were combined and reduced to 1 mL using N2	NR	[65]
Brooch	GC–MS	T	8	10	-	-	Phthalates, OPEs	4–7 d	NR	WB extracted in 30 mL acetonitrile using a wrist action shaker and reduced to 0.5 mL N2. Extracts were filtered through a 17 mm Teflon syringe filters with pore size of 0.2 μm	Personal low volume active air sampler	[46]
Wristband	GC–MS	T	5	11	-	-	PAHs, PCPs	4 d	Solvent conditioning with 3 × 1:1 ethyl acetate/hexane followed by 2 × 1:1 ethyl acetate/methanol on orbital shaker for > 2.5 h	WB rinsed 2 × with DI water and IPA. WB were extracted 2 × with ethyl acetate for 24 h. The extraction was repeated twice. Extracts were reduced to 0.5 mL and cleaned using C18 SPE with 4 mL 1:1 hexane:dichloromethane, and reduced to 1 mL prior to analysis	Portable online particle instrumentation, air quality sensors, personal environmental monitor, NO2 diffusion tubes, questionnaire	[92]
Wristband	GC–MS	T	24	19**	-	-	PCBs, PBDEs, OPFRs, NHFRs, pesticides	1 w	Solvent conditioning with 3 × 1:1 ethyl acetate/hexane followed by 2 × 1:1 ethyl acetate/methanol for > 2.5 h	1 g WB was extracted 2 × using 25 mL ethyl acetate for 2 h at 60 rpm. Extracts were combined and reduced to 300 μL and dilute with 3 mL AcCN. Extracts were loaded onto 500 mg C18 SPE cartridges; analytes were eluted with 6 mL AcCN (6 mL). Extracts were dried using N2 and reconstituted with 200 μL isooctane	Survey	[155]
Wristband	GC–MS	T	101	34	-	-	PBDEs, OPFRs, nBFRs	7 d	Solvent conditioning with 1:1 ethyl acetate/hexane followed by 1:1 ethyl acetate/methanol for 24 h	WB were extracted 2 × with 30 mL 1:1 hexane/acetone for 2 h. Extracts were combined, reduced with N2, and split volumetrically. The first aliquot was purified for PBDE and BFRs using neutral alumina, neutral silica gel, sulfuric acid–silica gel, and sodium sulfate. Analytes were eluted with 40 mL of DCM. The second aliquot for OPFRs and BEHTBP was purified using top neutral alumina, neutral silica, florisil, and sodium sulfate. Analytes were eluted using 40 mL of DCM and 40 mL of ethyl acetate. Each fraction was reduced to 1 mL prior to analyses	Stationary passive air samplers (PUF), thyroid function	[95]
Wristband, brooch	GC–MS	T	10	38	-	-	PAHs, PBDEs, nBFRs, OPEs	72 h	Solvent conditioning with 1:1 ethyl acetate/hexane and 1:1 ethyl acetate/methanol for 24 h	WB were extracted 2 × with 30 mL 1:1 hexane/acetone for 2 h while sonicating; WB were allowed to equilibrate in the solvent after the first extraction. Extracts were combined, reduced with N2 and split volumetrically. The first aliquot was purified for PBDE and BFRs using neutral alumina, neutral silica gel, sulfuric acid–silica gel, and sodium sulfate. Analytes were eluted with 40 mL of DCM. The second aliquot for PAHs, OPEs, and BEHTBP was purified using neutral alumina, neutral silica, florisil, and sodium sulfate. Analytes were eluted using 40 mL of DCM and 40 mL of ethyl acetate. Each fraction was reduced to 1 mL prior to analyses	Active air samples, hand wipes	[64]
Wristband	GC–MS	T	15	23	-	-	PBDEs, NBFRs, DPs, OPEs	24 h	Solvent conditioning with pentane for 3 days	WB were extracted in acetonitrile. Extracts were purified using florisil SPE cartridges rinsed with 8 mL of methanol and 4 mL of ethyl acetate. Analytes were eluted with 10 mL of ethyl acetate	T-shirts over work shirts (8–9 working h.)	[156]
Wristband	GC–MS	T	25	62		-	PAHs	7 d	Solvent conditioning with 3 × 1:1 ethyl acetate/hexane followed by 2 × 1:1 ethyl acetate/methanol on orbital shaker for > 2.5 h	WB extracted 2 × with 100 mL ethyl acetate at room temperature using at 60 RPMs. Extracts were reduced using N2	Interviews, temperature	[157]
Wristband	GC–MS	SS	246	-	191	-	Commercial chemicals, FRs, PAHs, PCBs, pesticides, phthalates, and EDCs	Unclear	WB rinsed with DI water and then baked at 300 °C for 180 min at 0.1 Torr. The vacuum oven was flushed with 99.99% nitrogen at 15, 30, 45, 60, 90, 120, and 180 min intervals during baking	WB were washed 2 × with DI water followed by isopropanol. Extraction was performed with two rounds of 100 mL ethyl acetate and reduced using. WB were purified using C18 SPE: extracts were first diluted to 3 mL with AcCN and loaded onto a pre-rinsed SPE cartridge. Analytes were eluted with 9 mL of AcCN	Questionnaire	[158]
Wristband and lapel	GC–MS	T	10	8	-	-	SVOCs, phthalates and alternatives, OPEs	Work shift	Solvent conditioning with 1:1 ethyl acetate/hexane followed by 1:1 ethyl acetate/methanol for 12 h	WB were extracted using 1:1 hexane:acetone for 12 h and concentrated using N2. Extracts were filtered with a 25 mm syringe filter with a 0.2 μm PTFE membrane, and cleaned using florisil SPE by eluting two separate fractions with 10 mL hexane, followed by 10 mL ethyl acetate. Samples were concentrated to 1 mL using N2 prior to analysis	Urine creatinine and biomarkers, questionnaire	[69]
Wristband	GC–MS	T	64	4	-	-	OPEs	7 d	WB conditioned with ethyl acetate, hexane, and methanol at 30 °C	Wristbands were extracted 2 × with 100 mL of ethyl acetate and reduced to 300 µL with N2. Three mL of AcCN was added to each sample, and extracts were loaded onto 500 mg C18 SPE pre-rinsed with 6 mL of AcCN. Analytes were eluted with 9 mL of AcCN. The extracts were reduced to 0.5 mL with N2 and solvent-exchanged to hexane prior to analysis	Urinary biomarkers, questionnaire	[159]
Wristband	GC–MS, GC-ECD	SS and T	69	16	106		Pesticides (fungicides, herbicides, insecticides), FRs, industrial compounds, plasticizers, PAHs, PCPs	30 d	Solvent conditioning with 3 × 1:1 ethyl acetate/hexane followed by 2 × 1:1 ethyl acetate/methanol on orbital shaker for > 2.5 h	WB were washed 2 × with DI water followed by isopropanol. WB were extracted 2 × with 100 mL of ethyl acetate for > 12 h at ambient temperature. The extracts were combined, and reduced to 1 mL with N2 prior to analysis	LDPE environmental passive sampling, survey	[67]
Wristband	GC–MS, GC-ECD	SS and T	92	64	17	-	Pesticides (fungicides, herbicides, insecticides)	7 d	WB rinsed with DI water and then baked at 300 °C for 180 min at 0.1 Torr. The vacuum oven was flushed with 99.99% nitrogen at 15, 30, 45, 60, 90, 120, and 180 min intervals during baking	WB were rinsed 2 × with DI water followed by isopropanol. WB were extracted 2 × with 100 mL ethyl acetate and reduced to 1 mL with N2. A 200 μL aliquot was purified using C18 SPE with acetonitrile and solvent exchanged to isooctane	Home survey, home pesticide inventory, agricultural area mapping, questionnaire	[71]
Wristband	GC–MS, GC-HRMS	T	77	23	-	-	OPEs, phthalates	7 d	Solvent conditioning with 1:1 ethyl acetate/hexane and 1:1 ethyl acetate/methanol for 24 h	WB were cut, weighed, and extracted 3 × with 10 mL 1:1 hexane/dichloromethane and then concentrated to 1.0 mL with N2. Extracts were fractionated using Florisil SPE cartridges, eluting the second fraction with 10 mL ethyl acetate for organophosphate esters and phthalates. The first and third fractions were eluted with 8 mL of hexane for the brominated flame retardants and with 8 mL of methanol for PFASs, respectively	Urinary biomarkers, hand wipes, dust	[75]
Wristband	GC–MS, GC-HRMS	T	72	49	-	-	OPEs, pesticides, phthalates, BFRs	7 d	Solvent conditioning with 1:1 ethyl acetate/hexane and 1:1 ethyl acetate/methanol for 24 h	WB extracted by sonicating 3 × with 10 mL hexane/dichloromethane for 15 min, and reduced to 1 mL with N2	Reporter gene assay in human kidney cells	[76]
Wristband and dog collar samplers	GC–MS, GC-HRMS	SS	30	66	-	-	PCBs, PBDEs, OPEs, common pollutants	5 d	Solvent conditioning with 1:1 ethyl acetate/hexane and 1:1 ethyl acetate/methanol for 24 h	Dog collars and wristband cut into pieces < 1.0 g, weighed, and extracted 3 × with 10 mL of a 50:50 (v/v) mixture of hexane/dichloromethane for 15 min with sonication. Extracts were concentrated to ∼1.0 mL with N2 and cleaned using deactivated florasil. Three fractions were collected, using hexane, ethyl acetate, and methanol. Fractions 1 and 2 were combined and concentrated to 1.0 mL using N2	Urinary biomarkers (dog and human), questionnaire, dog collar silicone samplers	[77]
Silicone dog tags	GC–MS, GC–MS/MS	SS, T	56	45	101	-	PBDEs and NHFRs, OPEs	30 d on-duty and 30 d off duty	Vacuum oven at 300 °C for 12 h at 0.1 Torr	Solvent extraction with 50 mL ethyl acetate (2 ×), N2 evaporation, SPE with acetonitrile, solvent exchange to isooctane	Survey	[70]
Wristband	GC–MS, GC–MS/MS	T	10	77	-	-	PBDEs, NHFRs, OPEs, PAHs	7 d	Solvent conditioning with 1:1 ethyl acetate/hexane and 1:1 ethyl acetate/methanol for 24 h	WB were extracted with 30 mL 1:1 hexane:acetone for 2 h in an ultrasonicating bath, and allowed to equilibrate for 12 h. The extract was removed, and the WB was extracted again with 20 mL in an ultrasonicating bath for 2 h. The extracts were combined, reduced to 1 mL using N2, solvent exchanged 2 × with hexane, and diluted to 4 mL. The extract samples were mixed for 30 s, and split into two equal samples for cleanup by multi-layer chromatography	-	[160]
Wristband	GC–MS, GC-QTOF	T & U	27	33	-	595 − 1011	PAHs, phthalates, phenols, chlorinated compounds, PCPs, combustion products, industrial products, plasticizers	5 d	WB rinsed with DI water and then baked at 300 °C for 180 min at 0.1 Torr. The vacuum oven was flushed with 99.99% nitrogen at 15, 30, 45, 60, 90, 120, and 180 min intervals during baking	WB were rinsed 2 × with DI water followed by IPA to remove solids or particles. WB were extracted 2 × with 100 mL of ethyl acetate for 2 h at 60 RPM. The two extracts were combined and reduced to < 1 mL with N2	Questionnaire	[78]
Wristband	GC–MS/MS	T	22	12	-	-	PCBs, pesticides, FR, PAHs, VOCs	2 d	WB rinsed with DI water and then baked at 300 °C for 180 min at 0.1 Torr. The vacuum oven was flushed with 99.99% nitrogen at 15, 30, 45, 60, 90, 120, and 180 min intervals during baking	WBs were washed 2 × with DI water followed by IPA. WB were extracted 2 × with 100 mL of ethyl acetate. The extracts were combined and reduced to 1 mL with N2	PUF active air monitoring sampler (backpack)	[44]
Wristband	GC–MS/MS	T	22	51	-	-	PAHs	2 d	Solvent conditioning with 1:1 ethyl acetate/hexane and 1:1 ethyl acetate/methanol for 24 h	WB were extracted 2 × with 100 mL ethyl acetate at room temperature at 60 RPM. Extracts were combined and reduced to 1 mL using N2	Active air monitoring with PUF in backpacks, urinary biomarkers	[45]
Wristband	GC–MS/MS	T	19	44**	-	-	PAHs	3 weeks	Solvent conditioning with 3 × 1:1 ethyl acetate/hexane followed by 2 × 1:1 ethyl acetate/methanol on orbital shaker for > 2.5 h	WBs were washed 2 × with DI water followed by IPA. WB were extracted 2 × with ethyl acetate and reduced to 1 mL using N2	Stationary LDPE samplers, daily exposure logs	[161]
Fresh Air sampler	GC-QTOF	T	33	22	-	-	VOCs and PAHs	4.3 d children	PSDs were first cleaned using hexane, and then baked at 300 °C for 130 min under N2	Thermal desorption	Ogawa pad, questionnaire	[62]
Wristband	GC/MS, LC–MS/MS	T	71	74	-	-	PBDEs, nBFRs, OPEs, (GC–MS); PAHs (LC-MSMS)	5 d	Solvent conditioning with 1:1 ethyl acetate/hexane and 1:1 ethyl acetate/methanol for 24 h	WB were extracted 2 × with 30 mL 1:1 hexane/acetone for 2 h while sonicating; WB were allowed to equilibrate in the solvent after the first extraction. Extracts were combined, reduced with N2 and split volumetrically. The first aliquot was purified for PBDE and BFRs using neutral alumina, neutral silica gel, sulfuric acid–silica gel, and sodium sulfate. Analytes were eluted with 40 mL of DCM. The second aliquot for PAHs, OPEs, and BEHTBP was purified using neutral alumina, neutral silica, florisil, and sodium sulfate. Analytes were eluted using 40 mL of DCM and 40 mL of ethyl acetate. Each fraction was reduced to 1 mL prior to analyses	Survey	[80]
Wristband	LC–MS	T	53	6	-	-	Nicotine, cotinine, TSNAs	7 d	Solvent conditioning with 3 × 1:1 ethyl acetate/hexane followed by 2 × 1:1 ethyl acetate/methanol on orbital shaker for > 2.5 h	Quick Easy Cheap Effective Rugged and Safe (QuEChERS) method	Urinary cotinine, interview, daily monitoring, air badges	[82]
Wristband	LC–MS/MS	T	31	1	-	-	Nicotine	7 d and 2 d	Solvent conditioning with 3 × 1:1 ethyl acetate/hexane followed by 2 × 1:1 ethyl acetate/methanol on orbital shaker for > 2.5 h	WB were treated with 4 mL of 0.1% formic acid and vortexed for 1 min. 1 mL of 1 M KOH was added and vortexed for 1 min. Each WB was then mixed with 3 mL AcCN for 5 min. The supernatant was mixed with 2 g MgSO4 and 0.5 g NaCL and mixed for 1 min. Extracts were centrifuged for 5 min, and 1 mL of supernatant was filtered with a PTFE syringe filter (13 mm diameter, 0.2 µm pore size)	Urinary cotinine, interview, questionnaire	[81]
Wristband	LC–MS/MS	T	230	7	-	-	Phenols	7 d	Solvent conditioning with 1:1 ethyl acetate/hexane and 1:1 ethyl acetate/methanol	WB were cut, accurately weighed, and extracted via sonication with 1:1 hexane/dichloromethane. Extracts were reduced to 1 mL using N2 and fractionated using florisil SPE cartridges. The second fraction was eluted using 10 mL ethyl acetate, first exchanged to hexane, and then methanol for LC–MS/MS analysis	House dust, hand wipes, urinary biomarkers, questionnaire	[83]
Wristband & stationary	LC–MS/MS	SS	30	0	31	-	PRs	5 d	Solvent conditioning with 1:1 ethyl acetate/hexane and 1:1 ethyl acetate/methanol for 30 min each	WB were extracted 3 × with 40 mL ethyl acetate with mechanical shaking for 30 min. The combined ethyl acetate extract was reduced at 40 °C with N2 to 200 μL. Prior to analysis, the extract was diluted to 400 μL with mobile phase (90:10 water/methanol containing 5 mM ammonium acetate) and adjusted to 1 mL with methanol	Questionnaire	[84]

^1^*T*, targeted analysis; *SS*, suspect screening; *U*, untargeted analysis

^2^Reported signals included both identified and unidentified signals

^3^Abbreviations include *VOCs*, volatile organic compounds; *OPP*, organophosphate pesticides; *Pyr*, pyrethroid insecticides; *OCP*, organochlorine pesticides; *OPEs*, organophosphate esters; *FRs*, flame retardants; *PAHs*, polycyclic aromatic hydrocarbons; *PCBs*, polychlorinated biphenyls; *SVOCs*, semi-volatile organic compounds; *NVOCs*, non-volatile organic compounds; *PBDEs/BDEs*, polybrominated diphenyl ethers; *OPFRs*, organophosphorus flame retardants; *BFRs*, brominated flame retardants; *PCPs*, personal care products; *NHFRs*, novel halogenated flame retardants; *nBFRs*, novel brominated flame retardants; *TSNAs*, tobacco-specific nitrosamines; *DPs*, dechlorane plus; *EDCs*, endocrine disrupting chemicals; *PRs*, plant protection products and biocidal active substances

^4^Silicone conditioning refers to the precleaning process to remove impurities and free siloxanes from the silicone matrix prior to deployment. Solvent conditioning use solvent-based extraction methods for preparing wristbands. Heat conditioning uses a combination of heat treatment and vacuum to remove free siloxanes

Of these studies, wristbands were the most commonly used PSD device, while a limited number used multiple placement strategies to isolate exposure pathways, including brooch samplers for airborne respiratory exposures, and isolated wristbands to minimize dermal contact [46, 61–65, 68, 69]. Silicone PSDs have been used to measure different classes of environmental pollutants, including polycyclic aromatic hydrocarbons (PAHS), brominated and organophosphate flame retardants (B- or OFR), pesticides and insecticides, phthalates, passive tobacco smoke exposure, and volatile organic chemicals (VOCs), among others. In most cases, PSDs exhibited high affinity for these chemical classes, highlighting the benefit of using this material for exposome monitoring. Most study participants wore wristbands for 7 days, with some studies extending to 30-day continuous wear periods [65, 67, 70].

Most PSDs (84%) were characterized using targeted approaches, where specific chemical classes were quantified with in-house analytical standards. An additional 18% of the studies included some form of suspect screening. Most exposures included volatile and semi-volatile compounds measured using gas-chromatography (GC), including single- and triple-quadrupole mass spectrometers, with some studies leveraging electron capture detectors for increasing specificity towards halogenated compounds [60, 67, 71, 72]. Only a few studies combined GC with HRMS, including time of flight (TOF) and Orbitrap mass spectrometers [61–63, 73–79]. Of the studies using HRMS technologies, only 50% were untargeted, defined as methods that used data-driven approaches for signal detection, filtering, and annotation. The use of liquid chromatography (LC) methods, which enables detection of many contemporary-use pesticides and emerging chemicals of concern, was also limited. Five studies included LC–MS analysis of wristbands, with one measuring pesticide exposures, one phenol exposure, one SVOCs, and the remaining two focused on passive tobacco smoke exposure [80–84]. None of these studies used LC-HRMS, a key technology for untargeted screening of many environmental exposures [85, 86].

Since the use of silicone PSDs is a new approach for passive exposure monitoring, 38 of the 44 reviewed studies combined PSDs with validated approaches for assessing exposures, including comparison to biomarker levels and established sampling devices. These include quantification of known exposure biomarkers in blood and urine [45, 68, 81, 87, 88], hand wipes [64, 75, 87], active air sampling [45, 46, 89, 90], and low-density polyethylene PSDs [67, 91], as well as using questionnaires to estimate past exposures [60, 61, 77, 78, 81, 82, 84, 87, 88, 92, 93]. Findings include a significant correlation between the PSD chemical concentrations and accepted biomarker measurements in urine [45, 87], demonstrating usability of silicone PSDs to evaluate personal exposures. These samplers similarly showed high specificity to detect unique chemical profiles, including detection of exposure profiles based on dietary and behavioral trends, as well as unique chemical signatures within different rooms of the same residence [84, 94].

Most of the reviewed studies include questionnaires to associate PSD detected chemical classes with behavioral, lifestyle, and demographic patterns that may influence exposure patterns and potential health outcomes. Although few studies focus on biological endpoints, recent applications have attempted to link PSD measurements to health outcomes, including DNA damage biomarkers [72], thyroid function [95], social behaviors in children [96], and respiratory-related disorders [62]. Interestingly, one study combined wristbands with effect-directed analysis (EDA) to identify wristband-captured exposures contributing to thyroid dysfunction [76]. In this study, extracted wristbands were tested using gene-reporter assays that evaluate thyroid disrupting bioactivity, providing a biological-based prioritization of compounds potentially contributing to adverse effects.

One of the challenges facing large-scale adoption of silicone-based PSDs for monitoring multiple exposures is uncertainty in partitioning and diffusion rates into the sampler matrix for compounds showing a wide range of physical–chemical properties [67, 91]. Contact with surfactants and oils, such as soaps and lotions, may also influence uptake of certain compounds. Since chemical uptake into wristbands vary, estimating environmental concentrations can be difficult [72]. To improve quantitative interpretation, recent efforts have focused on identifying partitioning coefficients by chemical class [44]. Silicone PSDs have been shown to outperform traditional sampler materials like low-density polyethylene (LDPE), showing improved sequestration of polar compounds and heavier polybrominated flame retardants [91]. However, other chemical classes have shown lower affinity for silicone, including PAHs [97]. While this could limit detection of important air- and smoke-related exposures, silicone showed a higher correlation with urinary PAH metabolites and outperformed polyurethane foam combined with active air sampling [45]. Sorbent bars coated in polydimethylsiloxane showed comparable performance for sampling for higher molecular weight PAHs, with stable uptake for periods greater than 24 h [62]. Additional chemical classes showing good affinity for PSDs include OFRs, compounds in tobacco smoke, and plasticizers [75, 87].

**Fig. 1:**
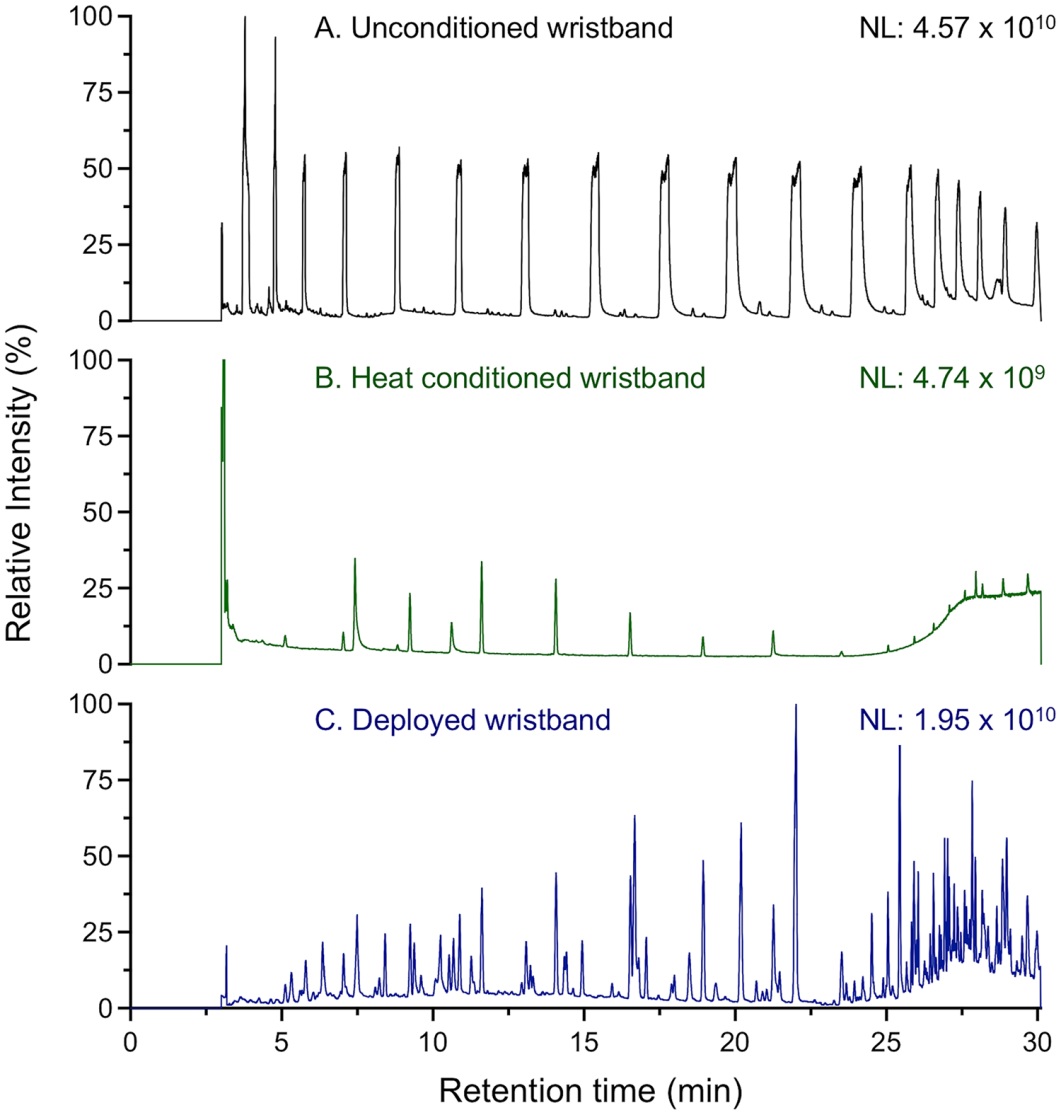
Sample total ion chromatograms for **A** unconditioned silicone wristband analyzed as received from the manufacturer; **B** silicone wristband conditioned for 18 h at 300 °C maintained at < 0.1 Torr with nitrogen venting at 15, 30, 45, 60, 90, 120, 180, 240, 300, and 360 min; **C** heat conditioned silicone wristband after a 7-day deployment period where the wristband was worn continuously by the study participant

PSDs are available commercially in a wide range of colors and sizes and can be modified to include text through embossing/debossing. While price varies depending on vendor, amount purchased, color/text options, and size, most are available at low cost (< $0.50 USD) and provide an economical solution for PSDs. However, wristbands purchased commercially often contain a high degree of impurities that can interfere with measurement sensitivity. Before deployment, thorough conditioning and cleaning are required to remove unbound siloxanes and other impurities [44, 65]. Dyes and inks can further contribute to background impurities, and testing should be performed to assess their impact before use. If available, uncolored or clear silicone materials should also be considered [68]. The importance of conditioning prior to wristband deployment is highlighted in Fig. 1. Uncleaned samplers result in a high degree of co-extracted siloxanes (Fig. 1A) that can impact compound detection, foul GC columns, and introduce a high degree of instrument contamination. Wristbands cleaned using solvent washing or heat treatment remove the majority of impurities present in the silicone (Fig. 1B), enhancing detection of exposures following wristband deployment (Fig. 1C). The most common method for silicone conditioning includes a series of washes using organic solvents, including ethyl acetate, methanol, hexanes, and pentane (Table 1). Wristbands are often equilibrated with each solvent for a period ranging from 30 min to multiple days [65, 87, 98]. Following all washes, wristbands are allowed to dry under an inert gas or in a clean environment prior to packaging for distribution.

While solvent washing can remove a significant number of impurities, this approach is expensive, time consuming, and difficult to adapt to large quantities. Recent attempts to lower time and cost of conditioning have been developed, including re-use of solvents and accelerated methods that reduce solvent-washing [44, 84]. Heat conditioning provides a suitable alternative to solvent washing and can result in considerable savings in personnel time and solvent costs [44]. When using heat conditioning, wristbands are heated to 250–300 °C and maintained under vacuum (< 1 Torr) with periodic nitrogen flushing. Heating times can vary, with some studies showing 3 h provides sufficient removal of siloxanes; experience with heat conditioned wristbands for untargeted analysis suggests periods of 20–24 h may be more suitable. Depending on the size of the oven, it is possible to prepare 50–100 wristbands per day. While heat conditioning improves wristband preparation throughput, effects of high temperatures on the silicone material, removal of less volatile siloxanes, and frequent cleaning of vacuum systems must be considered. All new batches of silicone must be tested for suitability with heat conditioning, as even minor changes in manufacturing can influence how silicone elastomers respond to the heating process. Cost-effective and robust conditioning of a large number of silicone PSDs is one of the main barriers for use in large population studies. While current conditioning approaches can increase costs by 50–1000-fold, continued development of heat conditioning and alternative strategies is expected to decrease cost and improve capacity.

Following deployment of silicone PSDs, exposure-related compounds must be removed from the matrix and transferred to a form that is amendable to the chosen analytical method. Since the majority of silicone PSDs have focused on volatile and semi-volatile exposures, most commonly used preparation methods are GC-friendly and include solvent extraction or thermal desorption (TD) (Table 1). The most common solvents for extraction include ethyl acetate, hexane, and dichloromethane. Extracts can then be processed through additional steps, including solvent evaporation and exchange, cleanup using solid-phase extraction (SPE), or injection as is. Care must be taken when selecting processing steps, as significant analyte loss could occur depending on the physiochemical properties of the analytes. For example, selection of extraction solvents with high octanol–water partitioning coefficients (*K*_*ow*_) may prevent extraction of more polar compounds, while drying steps can result in loss of volatile compounds.

TD methods have been used extensively for environmental sampling of volatile compounds, as well as analysis of silicone PSDs [44, 99]. When using TD to analyze PSDs, silicone samplers are heated so that compounds are volatized and either injected directly onto the GC column or trapped using filters prior to analysis. The advantages of these methods for deployed silicone samplers include improved sample preparation times, reduction in co-extracted matrix and non-volatiles that can foul GC systems, and the ability to characterize highly volatile organic compounds. Development of the Fresh Air wristband, which uses thermal desorption to analyze polydimethylsiloxane PSDs, shows it is possible to measure many different volatile and semi-volatile environmental exposures, and can be combined with untargeted analysis to identify exposures associated with health outcomes [61–63]. Although limited, LC analyses of wristbands have all used solvent extraction sample preparation methods, which is consistent with better detection of non-volatile and polar compounds [80–84].

## High-Resolution Mass Spectrometry for Measuring the External Exposome

Although PSDs have received considerable attention for exposure monitoring, their combination with untargeted analysis is limited and most applications have focused on measuring common classes of known exposures. These studies provide important insight into chemical exposures in human populations but do not allow detection of unsuspected or uncharacterized chemical that may be driving health effects [6, 100]. Untargeted analyses depend upon methods that use HRMS, as it enables sensitive detection of low-level chemicals while providing sufficient mass accuracy and resolution for prediction of chemical formulas [7]. One of the first studies to combine silicone PSDs with untargeted analysis used GCxGC-TOF to identify personal exposure pattern variation for 27 participants across multiple regions within an urban environment [78]. Wristbands were characterized using a combination of targeted and untargeted methods, with targeted analysis including semi-volatile organic compounds (SVOCs). Untargeted results showed variable exposure profiles that included up to 1,000 detected chemical features and identified distinct clusters of compounds that distinguished seasons and regions. Targeted SVOCs showed no difference among these regions, highlighting the importance of expanding beyond known exposures when considering variability in individual exposome profiles.

The Fresh Air wristband, which includes a polydimethylsiloxane coated sampler bar located in an enclosed PTFE chamber, has been used extensively with GC-based HRMS methods that include Orbitrap and QTOF-based analyses for sampling exposures [101]. Using untargeted analysis, Koelmel et al. incorporated stringent filtering and deconvolution strategies to detect and identify exposures, including up to 615 high confidence annotations from the original 6,000 chemical signals detected in samplers worn by participants enrolled in a study designed for identifying biomarkers of air pollution [63]. Due to their flexible sampler design, they have also shown that PSD placement, season, and residence type influence exposure profiles. Their work underscores the high sensitivity of PSDs when combined with untargeted analysis, and how this can be leveraged to characterize the external exposome and routes of exposure [61, 62].

Recently, ultra-high-resolution mass spectrometry methods have been applied to stationary silicone PSDs to evaluate microenvironment exposures, as well as silicone wristbands worn by study participants. In the study by Kalia et al., silicone samplers were placed throughout different rooms within a residential location for a period of 7 days and analyzed using untargeted GC-HRMS [94]. Although none of the detected chemical features were identified, 1,347 signals were measured across all samplers located within one residence after correcting for background using field blanks. Comparisons between rooms in the same residence using principal component analysis (PCA) showed detection of room-specific signatures was possible. Untargeted GC-HRMS has also used to characterize silicone wristbands worn by study participants; the results from this study show benefits of untargeted methods to characterize the exposome using PSDs, and demonstrate potential influence of sample and data processing on detected chemicals [74].

Many chemicals of emerging concern (CECs) are only detectable using LC-HRMS methods and are known to be volatilized and transported on aerosols; these include per- and polyfluorinated alkyl substances (PFAS). PFAS and many other CECs are ionizable, and partition between the gas and particulate phase depending on interaction with aqueous aerosols and matrices, resulting in unique interactions with PSDs dissimilar from nonpolar SVOCs and persistent organic pollutants [102]. Novel PSD designs have been developed to characterize PFAS and other target CECs in indoor air [103], outdoor air, [104], and aqueous matrices [105, 106]. Limited research has paired untargeted LC-HRMS analyses with PSD deployment of any kind [107], though active sampler applications with untargeted analysis show important airborne exposures from both biotic and abiotic sources can be detected using LC-HRMS and combined with biological endpoints, thus predicting disease risk [29, 108]. To date, untargeted LC-HRMS has not been used to characterize personal exposome profiles using wearable silicone PSDs, despite well-established instrumental protocols for detection of low abundance, polar environmental chemicals. This data gap highlights an opportunity for optimization of wearable PSDs for semi-polar and polar chemicals suitable for LC-HRMS analysis, potentially leveraging sorbent or material modifications and innovative untargeted analytical workflows.

## Analytical Considerations for Untargeted Analysis of Silicone PSDs

While targeted methods provide excellent sensitivity and can generate new insight into ongoing exposures, costs increase with the number of chemicals analyzed [109]. Thus, development of targeted exposome-level assays is cost-prohibitive and does not enable detection of unknown and uncharacterized exposures. Since untargeted analytical methods using HRMS maximize the number of chemicals that can be measured in a single sample, these approaches are optimal for combining the exposome with silicone PSDs. The most commonly used HRMS platforms include QTOFs, which estimate accurate mass based on the time an ion takes to traverse a given flight path, and Orbitraps, where injected ions are introduced into a charged and rotating spindle and the oscillation frequency of orbiting ions is used to estimate accurate mass. While both QTOFs and Orbitrap instruments have excellent mass accuracy for high-abundance peaks, Orbitraps that provide ultra-high-resolution capabilities (> 120,000) display the greatest sensitivity and resolution for low abundance environmental chemicals, making them the preferred platform for exposome research. Combined with adaptive algorithms for processing complex mass spectral data, it is now possible to detect over 100,000 chemical signals in samples, including low-level environmental pollutants [12, 110–112].

The number and types of chemicals detected in PSDs can be expanded by combining complementary separation and ionization approaches for HRMS. These include using alternate chromatography strategies, with LC and GC as the most comprehensive platforms for exposome-wide association studies. LC-HRMS platforms are best suited for measurement of polar molecules with ionizable functional groups, or large, non-polar molecules that include lipids, fatty acids, and sterols. However, many exposome chemicals are volatile enough to be introduced into the gas phase when heated and are not detected by LC-HRMS. Thus, GC-HRMS provides the best sensitivity and selectivity for these compounds [12, 113]. Most detected chemicals from wearable silicone PSDs will exhibit some degree of volatility and most exposures are best detected using GC-HRMS [78]. When analyzing silicone PSD extracts, care must be taken to ensure the extracted samples are suitable for the analytical method of choice. All analyses should use a rigorous QA/QC plan, including at least 10% of samples as field blanks, which are non-deployed PSDs that were subjected to similar storage and transportation conditions. Often, these blanks are needed to separate the background from true signals and can be used to filter silicone-related chemicals from the final results.

Following analysis of PSDs, chromatograms can be processed using a number of software tools. Key steps include identification of peaks and integration, deconvolution to identify mass spectra, and alignment of peaks across samples. Both commercial and open-source tools are available; however, algorithms optimized for detection of low abundance peaks are best for exposome research [114–117]. Deconvolution strategies enhance detection and identification of chemicals, with current approaches based upon peak shape similarity, hierarchical clustering, and correlation across samples [118–122]. By incorporating correlation for deconvolution, these methods are optimized for low abundance peaks that are often characterized by poor peak shape, and will include fragments, isotopes, and adducts from the same compound [123].

Identification of mass spectral signals is one of the key challenges in applying untargeted HRMS. Many detected ions do not match compounds listed in metabolomic or environmental chemical databases and authentic standards are not available. Computational approaches that assign annotation confidence can enhance prediction of chemical identities. These approaches harness multiple lines of evidence to evaluate the quality of annotation and, combined with appropriate databases, improve the number of annotated compounds [124, 125]. Multiple databases exist for exposome research, including the Blood-Exposome database [126] and CECScreen, which includes over 70,000 CECs and predicted metabolites [85, 126, 127]. The US EPA CompTox Dashboard provides a key resource for identifying detected chemicals [128], with information on 765,000 chemicals and includes in silico predicted electron ionization (GC) and MS/MS (LC) spectra for all entries [129, 130]. For annotating unknown peaks that do not match database entries, numerous tools can be used to characterize ion fragmentation patterns and predict possible identities and biotransformation products of parent metabolites [131, 132]. Continued efforts focused on developing new chemical databases that house both environmental chemicals and endogenous metabolites are expected to improve annotation capabilities for untargeted mass spectrometry data in exposome research [133]. Molecular networking of GC-HRMS spectra and MS/MS data from LC-HRMS [134, 135] provides an additional strategy for classifying and inferring potential chemical identities, thus enabling insight into related substructures and similar compounds based upon similarity networks among spectra.

## Exposomic Data Science

For exposome studies designed to evaluate a disease or other adverse outcomes, signals from untargeted profiling must be prioritized to identify which exposures are driving risk [59]. When applying an exposome-wide association study framework to study relationships between exposures and outcomes, uni- or multivariate data analysis approaches are applied to evaluate the relationship of each detected chemical with the outcome. Because identification of all detected signals is often not possible, variable selection enables prioritization of exposures for identification. Due to the large number of signals detected in exposome studies, traditional data analysis methods are challenged by false positives and robust identification of the top signals defining environment-disease relationships. There are several sources of error that lead to this issue, including insufficient sample size relative to the number of compounds analyzed, excessive false discovery rate from multiple hypothesis tests, and analyzing each part of known or hypothesized networks individually [136]. An alternative approach is to apply multivariate methods that analyze the entire HRMS dataset jointly. These methods represent the samples as points and determine projections of these points into lower dimensional space, hyperplanes, components, or latent variables, such that a measure of information about the data points is maximized.

Multivariate and data reduction analytic strategies solve two major issues with the traditional exposome-wide association studies by (1) increasing power, since corrections for multiple comparisons are performed on the number of latent features (tens) rather than the number of chemicals (hundreds or thousands), and (2) facilitating determination of networks, since the latent variables are constructed based upon statistical or functional similarity and jointly use information across chemicals. Linear versions of these methods, such as PCA, independent component analysis (ICA), canonical correlation analysis (CCA), linear discriminant analysis (LDA), and partial least squares discriminant analysis (PLS-DA), are popular due to their simplicity of interpretation [137–143]. Nonlinear methods, such as self-organizing maps, support vector machines, and random forests, are less useful for interpretation but can be more powerful than linear methods for regression or classification [144–146]. Continued development of multivariate and dimension reduction techniques for application to exposomic studies is an ongoing area of research, with future application of these methods being expected to reduce complexity of the exposome while improving insight into how chemical mixtures influence health. For further information about multivariate methods used in exposome applications, we refer the reader to the following review articles [147–149].

## Strategies for Operationalizing Deep Exposome Phenotyping

To realize measurement of the exposome, it is critical to consider exposures across multiple compartments. While most studies use HRMS to characterize the internal exposome using biological samples, personal PSDs provide complementary advantages. First, PSDs are much cheaper to produce and distribute compared with the cost for collecting blood or urine samples. Biologics often require special methods and trained personnel for collection, including clinical visits. These materials must be stored at low temperatures to maintain sample integrity, and different handling and storage procedures can increase variability. PSDs can be provided directly to participants and returned by mail, with limited-to-no contact between study participants and coordinators. This capability is especially important when considering the additional restrictions placed on in-person research due to the COVID-19 pandemic. Long-term, secure storage of biologics can also be costly, as storage in −80 °C is common. In contrast, PSDs can be stored in sealed bags at 4 °C or room temperature, since compounds are stable within the silicone matrix.

Although studies to date have only used silicone PSDs with a small number of participants (average 50 participants; max 255; Table 1), this technology has the potential to provide a key exposome measurement within longitudinal cohort studies. Using heat-based conditioning methods, silicone wristband PSDs can be produced for as little as $5–$10, which is significantly lower than the cost for clinical visits to complete blood or other biofluid collection. The low cost and non-invasive nature of the silicone PSDs allows routine distribution to participants in large cohorts at study enrollment, and additional PSDs can be provided to participants during longitudinal follow-up periods. While cost for analysis by untargeted HRMS can be in the $200–$500 range, longitudinal follow-up can prioritize participant selection based upon health outcomes. Finally, silicone PSDs may provide improved detection and reduced variability for exposures with short biological half-lives. Depending on the compound, time for clearance from the human body can vary on the range of days to decades. When measuring compounds with short biological half-lives in blood or urine, the ability to detect a biomarker is dependent on the time of sample collection. Thus, exposome measurements in biological samples often suffer from high variability for rapidly metabolized compounds [150]. Due to compound stability within the silicone matrix, PSDs eliminate biological transformation and enable detection of the parent compounds averaged over longer time scales [83].

**Fig. 2:**
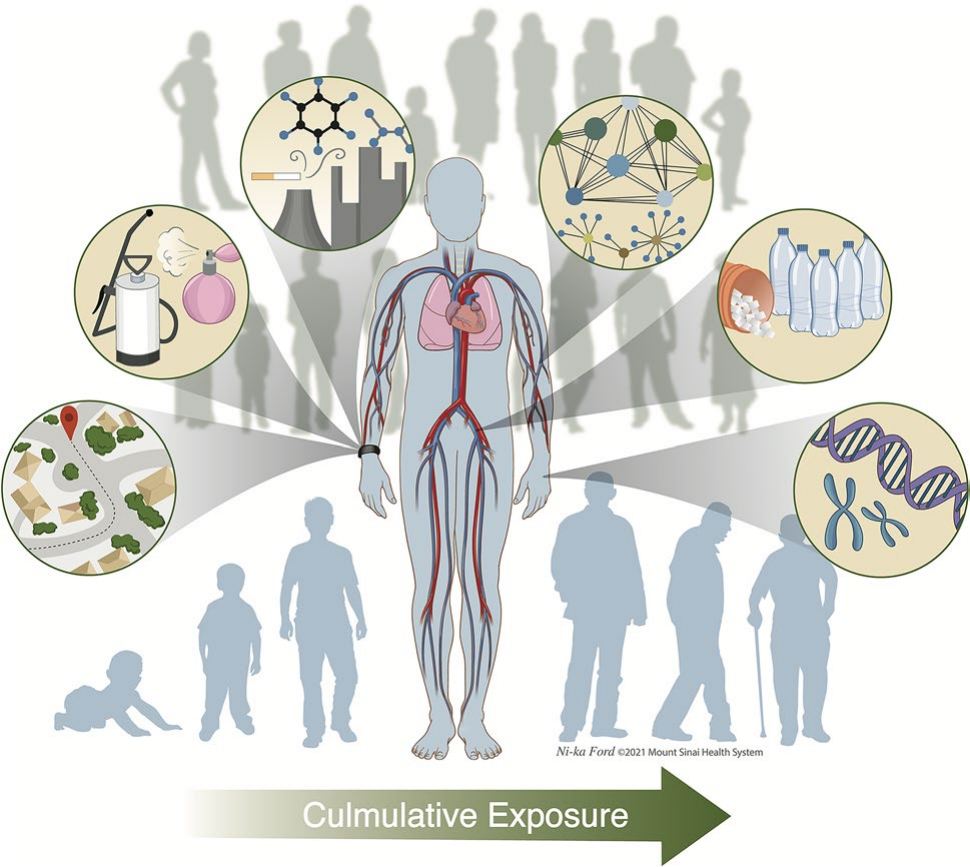
To better understand the human exposome, there is a need to measure exposures across both the external and internal exposome. Combining silicone PSDs, biological samples, and untargeted HRMS provides a unified strategy for deep exposome phenotyping that enables systematical measures of environmental exposures and corresponding biological exposures. While most efforts to date have focused on the internal exposome, silicone PSDs are low cost, non-invasive, easy to distribute, and allow measurement of compounds with short biological half-lives. Application of silicone PSDs within longitudinal studies will improve measurement of exposures at different life stages and provides the chemical coverage necessary for characterizing complex mixtures. Integrating external and internal measures of the exposome with other omic layers will allow a functional approach to understanding how environment contributes to disease risk, laying a foundation for the mechanisms underlying environment-related diseases

Due to their non-invasive nature, price, and ease of distribution, silicone PSDs are a key technology for measuring the exposome. While quantitative exposure measurements using PSDs are challenging if air-silicone partitioning behavior of analytes is not known, PSDs show considerable potential as a sampler to screen for the presence of both known and unexpected exposures that can be prioritized for further follow-up using traditional exposure assessment methods. Thus, rather than replacing collection of blood, urine, or other biological samples, they provide a complementary measure to assess specific compartments of the external exposome in population studies. For example, ingestion (eating and drinking) is one of the primary routes of environmental exposure and must be assessed using other approaches. Biospecimens also allow measurement of alterations across biological levels and long-term maladaptations, an important consideration for evaluating cumulative effects of environmental exposures. Combined with internal chemical and bioeffect monitoring, the use of silicone PSDs provides a strategy for deep exposome phenotyping in human populations (Fig. 2).

The goal of deep exposome phenotyping is to provide a systematic framework that operationalizes exposome-wide association studies of human health by combining the key measures necessary to understand the continuum from exposure to disease. Application in longitudinal cohorts can enable in-depth, comprehensive assessment of exposures, and when combined with untargeted HRMS analysis, provides the chemical coverage necessary for characterizing complex mixtures. Integrating external and internal measures of the exposome with multiple “-omic” layers will allow a functional approach to understanding how environment contributes to disease risk laying a foundation for the mechanisms underlying environment-related diseases [26, 27].

Because silicone PSDs are available at low cost, they can be easily incorporated into ongoing longitudinal studies and employed as a tool to estimate temporal changes in exposure patterns through repeated follow-up with new samplers. While the focus to date has been environmental health studies, silicone PSDs also provide a strategy for incorporating the exposome into precision medicine. Environmental factors are widely recognized for their potential to alter treatment efficacy and disease progression [151]. Silicone wristbands and other PSDs can provide a non-invasive means of chemical surveillance, helping identify patients for primary intervention or participants who would benefit from increased follow-up.

## Conclusions

While environment is one of the main drivers of disease risk, the ability to measure the complexity of the exposome is limited by its temporal nature, availability of samples, and the technology to detect complex exposures patterns. While considerable advances have been made in analytical strategies for the internal exposome, comparable methods for the external compartment are not well-developed. Silicone wristbands and other PSDs, which can be combined with untargeted HRMS platforms to characterize the exposome, are a natural way to integrate measures of the external exposome into longitudinal studies. These devices are cheap, non-invasive, and can be easily distributed. Previous studies demonstrate their suitability for many environmental chemical exposures, which is critical for success in exposome applications. By using untargeted approaches, it is possible to detect and identify ongoing exposures that may have not been expected or characterized, supporting pollution control and identification of the primary chemical exposures experienced by humans. Thus, continued development and application of silicone PSDs will facilitate greater understanding of how environmental exposures drive disease risk, while providing a feasible strategy for incorporating untargeted, high-resolution characterization of the external exposome in human studies.

## References

[1] Wild CP. Complementing the genome with an “exposome”: the outstanding challenge of environmental exposure measurement in molecular epidemiology. Cancer Epidemiol Biomarkers Prev. 2005;14(8):1847–1850. 10.1158/1055-9965.EPI-05-0456 16103423

[2] Rappaport SM, Smith MT. Epidemiology Environment and disease risks Science. 2010;330(6003):460–461. 10.1126/science.1192603 PMC4841276 20966241

[3] Rappaport SM. Implications of the exposome for exposure science. J Expo Sci Environ Epidemiol. 2011;21(1):5–9. 10.1038/jes.2010.50 21081972

[4] Miller GW, Jones DP. The nature of nurture: refining the definition of the exposome. Toxicol Sci. 2014;137(1):1–2. 10.1093/toxsci/kft251 PMC3871934 24213143

[5] Siroux V, Agier L, Slama R. The exposome concept: a challenge and a potential driver for environmental health research. Eur Respir Rev. 2016;25(140):124–129. 10.1183/16000617.0034-2016 PMC9487242 27246588

[6] Vermeulen R. The exposome and health: where chemistry meets biology. Sci. 2020;367(6476):392–396. 10.1126/science.aay3164 PMC7227413 31974245

[7] Uppal K. Computational metabolomics: a framework for the million metabolome. Chem Res Toxicol. 2016;29(12):1956–1975. 10.1021/acs.chemrestox.6b00179 PMC5376098 27629808

[8] Wild CP. The exposome: from concept to utility. Int J Epidemiol. 2012;41(1):24–32. 10.1093/ije/dyr236 22296988

[9] Park YH. High-performance metabolic profiling of plasma from seven mammalian species for simultaneous environmental chemical surveillance and bioeffect monitoring. Toxicol. 2012;295(1–3):47–55. 10.1016/j.tox.2012.02.007 PMC3332037 22387982

[10] Johnson JM. A practical approach to detect unique metabolic patterns for personalized medicine. Anal. 2010;135(11):2864–2870. 10.1039/c0an00333f PMC3069708 20838665

[11] Andra SS. Trends in the application of high-resolution mass spectrometry for human biomonitoring: an analytical primer to studying the environmental chemical space of the human exposome. Environ Int. 2017;100:32–61. 10.1016/j.envint.2016.11.026 PMC5322482 28062070

[12] Hu X. A scalable workflow to characterize the human exposome. Nat Commun. 2021;12(1):5575. 10.1038/s41467-021-25840-9 PMC8458492 34552080

[13] Soltow QA. High-performance metabolic profiling with dual chromatography-Fourier-transform mass spectrometry (DC-FTMS) for study of the exposome. Metabolomics. 2013;9(1 Suppl):S132–S143. 10.1007/s11306-011-0332-1 PMC4517297 26229523

[14] Stingone JA. Toward greater implementation of the exposome research paradigm within environmental epidemiology. Annu Rev Public Health. 2017;38:315–327. 10.1146/annurev-publhealth-082516-012750 PMC5664945 28125387

[15] Vineis P, et al. What is new in the exposome? Environ Int. 2020;143:105887. 10.1016/j.envint.2020.105887 32619912

[16] Miller G. Exposome: a new field, a new journal. Exposome. 2021;1(1). 10.1093/exposome/osab001 PMC10399727 37538531

[17] Jin L. Use of untargeted metabolomics to explore the air pollution-related disease continuum. Curr Environ Health Rep. 2021;8(1):7–22. 10.1007/s40572-020-00298-x 33420964

[18] Li S. Understanding mixed environmental exposures using metabolomics via a hierarchical community network model in a cohort of California women in 1960’s. Reprod Toxicol. 2020;92:57–65. 10.1016/j.reprotox.2019.06.013 PMC6949431 31299210

[19] Nassan FL. Metabolomic signatures of the long-term exposure to air pollution and temperature. Environ Health. 2021;20:1–3. 10.1186/s12940-020-00683-x PMC7788989 33413450

[20] Bonvallot N. Metabolomics as a powerful tool to decipher the biological effects of environmental contaminants in humans. Curr Opin Toxicol. 2018;8:48–56.

[21] Bonvallot N, et al. Metabolomics tools for describing complex pesticide exposure in pregnant women in Brittany (France). PLoS One. 2013;8(5):e64433. 10.1371/journal.pone.0064433 PMC3660334 23704985

[22] Fischer ST. Low-level maternal exposure to nicotine associates with significant metabolic perturbations in second-trimester amniotic fluid. Environ Int. 2017;107:227–234. 10.1016/j.envint.2017.07.019 PMC5569895 28759762

[23] Walker DI. High-resolution metabolomics of occupational exposure to trichloroethylene. Int J Epidemiol. 2016;45(5):1517–1527. 10.1093/ije/dyw218 PMC5100622 27707868

[24] Lu Y. Mass spectrometry-based metabolomics reveals occupational exposure to per- and polyfluoroalkyl substances relates to oxidative stress, fatty acid beta-oxidation disorder, and kidney injury in a manufactory in China. Environ Sci Technol. 2019;53(16):9800–9809. 10.1021/acs.est.9b01608 31246438

[25] Niedzwiecki MM. High-resolution metabolomic profiling of Alzheimer’s disease in plasma. Ann Clin Transl Neurol. 2020;7(1):36–45. 10.1002/acn3.50956 PMC6952314 31828981

[26] Jeong A. Perturbation of metabolic pathways mediates the association of air pollutants with asthma and cardiovascular diseases. Environ Int. 2018;119:334–345. 10.1016/j.envint.2018.06.025 29990954

[27] Chadeau-Hyam M. Meeting-in-the-middle using metabolic profiling—a strategy for the identification of intermediate biomarkers in cohort studies. Biomarkers. 2011;16(1):83–88. 10.3109/1354750X.2010.533285 21114379

[28] Chang CJ, et al. Per- and polyfluoroalkyl substance (PFAS) exposure, maternal metabolomic perturbation, and fetal growth in African American women: a meet-in-the-middle approach. Environ Int. 2021;158:106964. 10.1016/j.envint.2021.106964 PMC8688254 34735953

[29] Jiang C, et al. Dynamic human environmental exposome revealed by longitudinal personal monitoring. Cell. 2018;175(1):277–291 e31. 10.1016/j.cell.2018.08.060 PMC6472932 30241608

[30] Kloog I, et al. A new hybrid spatio-temporal model for estimating daily multi-year PM2.5 concentrations across northeastern USA using high resolution aerosol optical depth data. Atmos Environ (1994). 2014;95:581–590. 10.1016/j.atmosenv.2014.07.014 PMC5621749 28966552

[31] Halonen JI. Urban air pollution, and asthma and COPD hospital emergency room visits. Thorax. 2008;63(7):635–641. 10.1136/thx.2007.091371 18267984

[32] Peacock JL. Outdoor air pollution and respiratory health in patients with COPD. Thorax. 2011;66(7):591–596. 10.1136/thx.2010.155358 21459856

[33] Hyder A, et al. PM2.5 exposure and birth outcomes: use of satellite- and monitor-based data. Epidemiol. 2014;25(1):58–67. 10.1097/EDE.0000000000000027 PMC4009503 24240652

[34] VoPham T, et al. Dioxin exposure and breast cancer risk in a prospective cohort study. Environ Res. 2020;186:109516. 10.1016/j.envres.2020.109516 PMC7363533 32305677

[35] Zhang Z. Remote sensing and disease control in China: past, present and future Parasit Vectors. 2013;6:11. 10.1186/1756-3305-6-11 PMC3558403 23311958

[36] Sorek-Hamer M, Just AC, Kloog I. Satellite remote sensing in epidemiological studies. Curr Opin Pediatr. 2016;28(2):228–234. 10.1097/MOP.0000000000000326 PMC4844834 26859287

[37] VoPham T. Linking pesticides and human health: a geographic information system (GIS) and Landsat remote sensing method to estimate agricultural pesticide exposure Appl Geogr. 2015;62:171–181. 10.1016/j.apgeog.2015.04.009 PMC5580968 28867851

[38] Ebhuoma O, Gebreslasie M. Remote sensing-driven climatic/environmental variables for modelling malaria transmission in sub-Saharan Africa. Int J Environ Res Public Health. 2016;13(6). 10.3390/ijerph13060584 PMC4924041 27314369

[39] Hunter PD. Using remote sensing to aid the assessment of human health risks from blooms of potentially toxic cyanobacteria. Environ Sci Technol. 2009;43(7):2627–2633. 10.1021/es802977u 19452927

[40] Hay SI. An overview of remote sensing and geodesy for epidemiology and public health application. Adv Parasitol. 2000;47:1–35. 10.1016/s0065-308x(00)47005-3 PMC3164799 10997203

[41] Fletcher-Lartey SM, Caprarelli G. Application of GIS technology in public health: successes and challenges. Parasitol. 2016;143(4):401–415. 10.1017/S0031182015001869 26831619

[42] Seltenrich N. Remote-sensing applications for environmental health research. Environ Health Perspect. 2014;122(10):A268–A275. 10.1289/ehp.122-A268 PMC4181909 25272250

[43] Zhang P. Defining the scope of exposome studies and research needs from a multidisciplinary perspective. Environ Sci Technol Lett. 2021;8(10):839–852. 10.1021/acs.estlett.1c00648 PMC8515788 34660833

[44] Anderson KA. Preparation and performance features of wristband samplers and considerations for chemical exposure assessment. J Expo Sci Environ Epidemiol. 2017;27(6):551–559. 10.1038/jes.2017.9 PMC5658681 28745305

[45] Dixon HM. Silicone wristbands compared with traditional polycyclic aromatic hydrocarbon exposure assessment methods. Anal Bioanal Chem. 2018;410(13):3059–3071. 10.1007/s00216-018-0992-z PMC5910488 29607448

[46] Okeme JO. Polydimethylsiloxane (silicone rubber) brooch as a personal passive air sampler for semi-volatile organic compounds. Chemosphere. 2018;208:1002–1007. 10.1016/j.chemosphere.2018.05.196 30068024

[47] Mason JB. Evaluation of passive samplers for assessment of community exposure to toxic air contaminants and related pollutants. Environ Sci Technol. 2011;45(6):2243–2249. 10.1021/es102500v 21322547

[48] Clinkenbeard RE. A field comparison of the IOM inhalable aerosol sampler and a modified 37-mm cassette. Appl Occup Environ Hyg. 2002;17(9):622–627. 10.1080/10473220290095943 12216591

[49] Shirdel M. Passive personal air sampling of dust in a working environment—a pilot study. J Occup Environ Hyg. 2019;16(10):675–684. 10.1080/15459624.2019.1648814 31442106

[50] Hansen J. Nitrous oxide exposure among dental personnel and comparison of active and passive sampling techniques. Ann Work Expo Health. 2019;63(3):337–348. 10.1093/annweh/wxz003 30855661

[51] Bohlin P, Jones KC, Strandberg B. Occupational and indoor air exposure to persistent organic pollutants: a review of passive sampling techniques and needs. J Environ Monit. 2007;9(6):501–509. 10.1039/b700627f 17554420

[52] Gibbs JL. Passive sampling for indoor and outdoor exposures to chlorpyrifos, azinphos-methyl, and oxygen analogs in a rural agricultural community. Environ Health Perspect. 2017;125(3):333–341. 10.1289/EHP425 PMC5332193 27517732

[53] Strandberg B. Evaluation of polyurethane foam passive air sampler (PUF) as a tool for occupational PAH measurements. Chemosphere. 2018;190:35–42. 10.1016/j.chemosphere.2017.09.106 28985535

[54] Lan Q. Hematotoxicity in workers exposed to low levels of benzene. Science. 2004;306 (5702):1774–1776. 10.1126/science.1102443 PMC1256034 15576619

[55] O’Connell SG, Anderson KA, Epstein MI. Determining chemical air equivalency using silicone personal monitors. J Expo Sci Environ Epidemiol. 2021. 10.1038/s41370-021-00332-6 PMC8920887 33953340

[56] Jamin EL. Untargeted profiling of pesticide metabolites by LC-HRMS: an exposomics tool for human exposure evaluation. Anal Bioanal Chem. 2014;406(4):1149–1161. 10.1007/s00216-013-7136-2 23892877

[57] Bessonneau V, Pawliszyn J, Rappaport SM. The saliva exposome for monitoring of individuals’ health trajectories. Environ Health Perspect. 2017;125(7):077014. 10.1289/EHP1011 PMC5801473 28743678

[58] Ladva CN, et al. Metabolomic profiles of plasma, exhaled breath condensate, and saliva are correlated with potential for air toxics detection. J Breath Res. 2017;12(1):016008. 10.1088/1752-7163/aa863c PMC5803078 28808178

[59] Jones DP. Sequencing the exposome: a call to action. Toxicol Rep. 2016;3:29–45. 10.1016/j.toxrep.2015.11.009 PMC4692045 26722641

[60] Donald CE, et al. Silicone wristbands detect individuals’ pesticide exposures in West Africa. R Soc Open Sci. 2016;3(8):160433. 10.1098/rsos.160433 PMC5108971 27853621

[61] Koelmel JP. Head, shoulders, knees, and toes: placement of wearable passive samplers alters exposure profiles observed. Environ Sci Technol. 2021;55(6):3796–3806. 10.1021/acs.est.0c05522 33625210

[62] Lin EZ. The Fresh Air wristband: a wearable air pollutant sampler. Environ Sci Technol Lett. 2020;7(5):308–314.

[63] Koelmel JP, et al. Exploring the external exposome using wearable passive samplers—the China BAPE study. Environ Pollut. 2021;270:116228. 10.1016/j.envpol.2020.116228 33360595

[64] Wang S, et al. Silicone wristbands integrate dermal and inhalation exposures to semi-volatile organic compounds (SVOCs). Environ Int. 2019;132:105104. 10.1016/j.envint.2019.105104 PMC6774250 31465955

[65] O’Connell SG, Kincl LD, Anderson KA. Silicone wristbands as personal passive samplers. Environ Sci Technol. 2014;48(6):3327–3335. 10.1021/es405022f PMC3962070 24548134

[66] Hamzai L, et al. A systematic review of the use of silicone wristbands for environmental exposure assessment, with a focus on polycyclic aromatic hydrocarbons (PAHs). J Expo Sci Environ Epidemiol. 2021. 10.1038/s41370-021-00359-9 34302044

[67] Bergmann AJ. Multi-class chemical exposure in rural Peru using silicone wristbands. J Expo Sci Environ Epidemiol. 2017;27(6):560–568. 10.1038/jes.2017.12 PMC5658680 28745304

[68] Nguyen LV. Can silicone passive samplers be used for measuring exposure of e-waste workers to flame retardants? Environ Sci Technol. 2020;54(23):15277–15286. 10.1021/acs.est.0c05240 33196172

[69] Craig JA. Exposure of nail salon workers to phthalates, di(2-ethylhexyl) terephthalate, and organophosphate esters: a pilot study. Environ Sci Technol. 2019;53(24):14630–14637. 10.1021/acs.est.9b02474 PMC7192361 31736299

[70] Poutasse CM, et al. Discovery of firefighter chemical exposures using military-style silicone dog tags. Environ Int. 2020;142:105818. 10.1016/j.envint.2020.105818 PMC9985454 32521346

[71] Harley KG. Determinants of pesticide concentrations in silicone wristbands worn by Latina adolescent girls in a California farmworker community: the COSECHA youth participatory action study. Sci Total Environ. 2019;652:1022–1029. 10.1016/j.scitotenv.2018.10.276 PMC6309742 30380470

[72] Vidi PA. Personal samplers of bioavailable pesticides integrated with a hair follicle assay of DNA damage to assess environmental exposures and their associated risks in children. Mutat Res. 2017;822:27–33. 10.1016/j.mrgentox.2017.07.003 PMC5607735 28844239

[73] Reddam A, et al. Longer commutes are associated with increased human exposure to tris(1,3-dichloro-2-propyl) phosphate. Environ Int. 2020;136:105499. 10.1016/j.envint.2020.105499 PMC7061053 31999975

[74] Travis SC, Kordas K, Aga DS. Optimized workflow for unknown screening using gas chromatography high-resolution mass spectrometry expands identification of contaminants in silicone personal passive samplers. Rapid Commun Mass Spectrom. 2021;35(8):e9048. 10.1002/rcm.9048 33444483

[75] Hammel SC. Comparing the use of silicone wristbands, hand wipes, and dust to evaluate children’s exposure to flame retardants and plasticizers. Environ Sci Technol. 2020;54(7):4484–4494. 10.1021/acs.est.9b07909 PMC7430043 32122123

[76] Kassotis CD. Thyroid receptor antagonism of chemicals extracted from personal silicone wristbands within a papillary thyroid cancer pilot study. Environ Sci Technol. 2020;54(23):15296–15312. 10.1021/acs.est.0c05972 PMC7819617 33185092

[77] Wise CF. Comparative exposure assessment using silicone passive samplers indicates that domestic dogs are sentinels to support human health research. Environ Sci Technol. 2020;54(12):7409–7419. 10.1021/acs.est.9b06605 PMC7655112 32401030

[78] Manzano CA. Patterns of personal exposure to urban pollutants using personal passive samplers and GC x GC/ToF-MS. Environ Sci Technol. 2019;53(2):614–624. 10.1021/acs.est.8b06220 30575390

[79] Roodt AP. Human skin volatiles: passive sampling and GCxGC-ToFMS analysis as a tool to investigate the skin microbiome and interactions with anthropophilic mosquito disease vectors. J Chromatogr B Analyt Technol Biomed Life Sci. 2018;1097–1098:83–93. 10.1016/j.jchromb.2018.09.002 30212730

[80] Wang S, et al. The use of silicone wristbands to evaluate personal exposure to semi-volatile organic chemicals (SVOCs) in France and Italy. Environ Pollut. 2020;267:115490. 10.1016/j.envpol.2020.115490 33254690

[81] Quintana PJE. Nicotine levels in silicone wristband samplers worn by children exposed to secondhand smoke and electronic cigarette vapor are highly correlated with child’s urinary cotinine. J Expo Sci Environ Epidemiol. 2019;29(6):733–741. 10.1038/s41370-019-0116-7 30728487

[82] Quintana PJE. Nicotine, cotinine, and tobacco-specific nitrosamines measured in children’s silicone wristbands in relation to secondhand smoke and E-cigarette vapor exposure. Nicotine Tob Res. 2021;23(3):592–599. 10.1093/ntr/ntaa140 PMC8248526 33009807

[83] Levasseur JL, et al. Young children’s exposure to phenols in the home: associations between house dust, hand wipes, silicone wristbands, and urinary biomarkers. Environ Int. 2021;147:106317. 10.1016/j.envint.2020.106317 PMC7856225 33341585

[84] Aerts R. Silicone wristband passive samplers yield highly individualized pesticide residue exposure profiles. Environ Sci Technol. 2018;52(1):298–307. 10.1021/acs.est.7b05039 29185731

[85] Meijer J, et al. An annotation database for chemicals of emerging concern in exposome research. Environ Int. 2021;152:106511. 10.1016/j.envint.2021.106511 33773387

[86] Getzinger G, Ferguson P. Illuminating the exposome with high-resolution accurate-mass mass spectrometry and nontargeted analysis. Curr Opin Environ Sci Health. 2020;15:49–56.

[87] Hammel SC. Measuring personal exposure to organophosphate flame retardants using silicone wristbands and hand wipes. Environ Sci Technol. 2016;50(8):4483–4491. 10.1021/acs.est.6b00030 PMC4872512 26975559

[88] Hammel SC. Evaluating the use of silicone wristbands to measure personal exposure to brominated flame retardants. Environ Sci Technol. 2018;52(20):11875–11885. 10.1021/acs.est.8b03755 PMC6445795 30216050

[89] Okeme JO. Calibration of polydimethylsiloxane and XAD-Pocket passive air samplers (PAS) for measuring gas- and particle-phase SVOCs. Atmos Environ. 2016;143:202–208.

[90] Okeme JO. Passive air sampling of flame retardants and plasticizers in Canadian homes using PDMS, XAD-coated PDMS and PUF samplers. Environ Pollut. 2018;239:109–117. 10.1016/j.envpol.2018.03.103 29649757

[91] Allan IJ. Passive sampling for target and nontarget analyses of moderately polar and nonpolar substances in water. Environ Toxicol Chem. 2013;32(8):1718–1726. 10.1002/etc.2260 23625759

[92] Reche C, et al. Athletes’ exposure to air pollution during World Athletics Relays: a pilot study. Sci Total Environ. 202;717:137161. 10.1016/j.scitotenv.2020.137161 32065890

[93] Hendryx M, et al. Personal exposure to polycyclic aromatic hydrocarbons in Appalachian mining communities. Environ Pollut. 2020;257:113501. 10.1016/j.envpol.2019.113501 PMC6981027 31706774

[94] Kalia V. Unsupervised dimensionality reduction for exposome research Curr Opin Environ Sci Health. 2020;15:32–38. 10.1016/j.coesh.2020.05.001 PMC7467332 32905218

[95] Wang S. Association between thyroid function and exposures to brominated and organophosphate flame retardants in rural Central Appalachia. Environ Sci Technol. 2020;54(1):325–334. 10.1021/acs.est.9b04892 31820947

[96] Lipscomb ST. Cross-sectional study of social behaviors in preschool children and exposure to flame retardants. Environ Health. 2017;16:1–23. 10.1186/s12940-017-0224-6 PMC5343384 28274271

[97] O’Connell SG. Improvements in pollutant monitoring: optimizing silicone for co-deployment with polyethylene passive sampling devices. Environ Pollut. 2014;193:71–78. 10.1016/j.envpol.2014.06.019 PMC4140445 25009960

[98] Kile ML. Using silicone wristbands to evaluate preschool children’s exposure to flame retardants. Environ Res. 2016;147:365–372. 10.1016/j.envres.2016.02.034 PMC4821754 26945619

[99] Dettmer K, Engewald W. Adsorbent materials commonly used in air analysis for adsorptive enrichment and thermal desorption of volatile organic compounds. Anal Bioanal Chem. 2002;373(6):490–500. 10.1007/s00216-002-1352-5 12172684

[100] Escher BI, Stapleton HM, Schymanski EL. Tracking complex mixtures of chemicals in our changing environment. Sci. 2020;367(6476):388–392. 10.1126/science.aay6636 PMC7153918 31974244

[101] Doherty BT. Use of exposomic methods incorporating sensors in environmental epidemiology. Curr Environ Health Rep. 2021;8(1):34–41. 10.1007/s40572-021-00306-8 33569731

[102] Lai FY, et al. A critical review on passive sampling in air and water for per- and polyfluoroalkyl substances (PFASs). TrAC Trends in Analytical Chemistry. 2019;121.

[103] Winkens K. Perfluoroalkyl acids and their precursors in indoor air sampled in children’s bedrooms. Environ Pollut. 2017;222:423–432. 10.1016/j.envpol.2016.12.010 28012670

[104] Dixon-Anderson E, Lohmann R. Field-testing polyethylene passive samplers for the detection of neutral polyfluorinated alkyl substances in air and water. Environ Toxicol Chem. 2018;37(12):3002–3010. 10.1002/etc.4264 PMC6350245 30395357

[105] Kaserzon SL. Calibration and validation of a novel passive sampling device for the time integrative monitoring of per- and polyfluoroalkyl substances (PFASs) and precursors in contaminated groundwater. J Hazard Mater. 2019;366:423–431. 10.1016/j.jhazmat.2018.12.010 30554088

[106] Gobelius L. Calibration and application of passive sampling for per- and polyfluoroalkyl substances in a drinking water treatment plant. J Hazard Mater. 2019;362:230–237. 10.1016/j.jhazmat.2018.09.005 30240997

[107] Soulier C, Coureau C, Togola A. Environmental forensics in groundwater coupling passive sampling and high resolution mass spectrometry for screening. Sci Total Environ. 2016;563–564:845–854. 10.1016/j.scitotenv.2016.01.056 26803221

[108] Jiang C. Decoding personal biotic and abiotic airborne exposome. Nat Protoc. 2021;16(2):1129–1151. 10.1038/s41596-020-00451-8 33437065

[109] Walker DI, et al. eds. Population screening for biological and environmental properties of the human metabolic phenotype: implications for personalized medicine. Metabolic Phenotyping in Personalized and Public Healthcare, ed. J.K. Nicholson, et al. 2016. Elsevier.

[110] Go YM. Reference standardization for mass spectrometry and high-resolution metabolomics applications to exposome research. Toxicol Sci. 2015;148(2):531–543. 10.1093/toxsci/kfv198 PMC4675836 26358001

[111] Walker DI. The metabolome: a key measure for exposome research in epidemiology. Current Epidemiology Reports. 2019;6(2):93–103. PMC6905435 31828002

[112] Walker DI, et al. High-resolution exposomics and metabolomics reveals specific associations in cholestatic liver diseases. Hepatol Commun. 2021. 10.1002/hep4.1871 PMC9035559 34825528

[113] Manz KE, et al. Targeted and non-targeted detection and characterization of trace organic chemicals in human serum and plasma using QuEChERS extraction. Toxicol Sci. 2021. 10.1093/toxsci/kfab121 PMC8714361 34668567

[114] Uppal K. xMSanalyzer: automated pipeline for improved feature detection and downstream analysis of large-scale, non-targeted metabolomics data. BMC Bioinformatics. 2013;14:15. 10.1186/1471-2105-14-15 PMC3562220 23323971

[115] Yu T. apLCMS—adaptive processing of high-resolution LC/MS data. Bioinformatics. 2009;25(15):1930–1936. 10.1093/bioinformatics/btp291 PMC2712336 19414529

[116] Yu T, Jones DP. Improving peak detection in high-resolution LC/MS metabolomics data using preexisting knowledge and machine learning approach. Bioinformatics. 2014;30(20):2941–2948. 10.1093/bioinformatics/btu430 PMC4184266 25005748

[117] Smith CA. XCMS: processing mass spectrometry data for metabolite profiling using nonlinear peak alignment, matching, and identification. Anal Chem. 2006;78(3):779–787. 10.1021/ac051437y 16448051

[118] Smirnov A, et al. ADAP-GC 3.2: graphical software tool for efficient spectral deconvolution of gas chromatography-high-resolution mass spectrometry metabolomics data. J Proteome Res. 2018;17(1):470–478. 10.1021/acs.jproteome.7b00633 29076734

[119] Smirnov A, et al. ADAP-GC 4.0: application of clustering-assisted multivariate curve resolution to spectral deconvolution of gas chromatography-mass spectrometry metabolomics data. Anal Chem. 2019;91(14):9069–9077. 10.1021/acs.analchem.9b01424 PMC6705124 31274283

[120] Broeckling CD. RAMClust: a novel feature clustering method enables spectral-matching-based annotation for metabolomics data. Anal Chem. 2014;86(14):6812–6817. 10.1021/ac501530d 24927477

[121] Yao L. Data processing for GC-MS- and LC-MS-based untargeted metabolomics. Methods Mol Biol. 2019;1978:287–299. 10.1007/978-1-4939-9236-2_18 31119670

[122] Sapozhnikova Y. Non-targeted screening of chemicals migrating from paper-based food packaging by GC-Orbitrap mass spectrometry. Talanta. 2021;226:122120. 10.1016/j.talanta.2021.122120 33676675

[123] Kachman M. Deep annotation of untargeted LC-MS metabolomics data with Binner. Bioinformatics. 2020;36(6):1801–1806. 10.1093/bioinformatics/btz798 PMC7828469 31642507

[124] Uppal K, Walker DI, Jones DP. xMSannotator: an R package for network-based annotation of high-resolution metabolomics data. Anal Chem. 2017;89(2):1063–1067. 10.1021/acs.analchem.6b01214 PMC5447360 27977166

[125] Nash WJ, Dunn WB. From mass to metabolite in human untargeted metabolomics: recent advances in annotation of metabolites applying liquid chromatography-mass spectrometry data. TrAC Trends Anal Chem. 2018.

[126] Barupal DK, Fiehn O. Generating the blood exposome database using a comprehensive text mining and database fusion approach. Environ Health Perspect. 2019;127(9):97008. 10.1289/EHP4713 PMC6794490 31557052

[127] Pourchet M, et al. Suspect and non-targeted screening of chemicals of emerging concern for human biomonitoring, environmental health studies and support to risk assessment: from promises to challenges and harmonisation issues. Environ Int. 2020;139:105545. 10.1016/j.envint.2020.105545 32361063

[128] Williams AJ. The CompTox Chemistry Dashboard: a community data resource for environmental chemistry. J Cheminform. 2017;9(1):61. 10.1186/s13321-017-0247-6 PMC5705535 29185060

[129] McEachran AD. “MS-Ready” structures for non-targeted high-resolution mass spectrometry screening studies. J Cheminform. 2018;10(1):45. 10.1186/s13321-018-0299-2 PMC6117229 30167882

[130] McEachran AD. Linking in silico MS/MS spectra with chemistry data to improve identification of unknowns. Sci Data. 2019;6(1):141. 10.1038/s41597-019-0145-z PMC6677792 31375670

[131] Ruttkies C, Neumann S, Posch S. Improving MetFrag with statistical learning of fragment annotations. BMC Bioinformatics. 2019;209(1):376. 10.1186/s12859-019-2954-7 PMC6612146 31277571

[132] Duhrkop K. Searching molecular structure databases with tandem mass spectra using CSI:FingerID. Proc Natl Acad Sci U S A. 2015;112(41):12580–12585. 10.1073/pnas.1509788112 PMC4611636 26392543

[133] Vinaixa M. Mass spectral databases for LC/MS- and GC/MS-based metabolomics: state of the field and future prospects. TrAC Trends Anal Chem. 2016;78:23–35.

[134] Yang JY. Molecular networking as a dereplication strategy. J Nat Prod. 2013;76(9):1686–1699. 10.1021/np400413s PMC3936340 24025162

[135] Aksenov AA. Auto-deconvolution and molecular networking of gas chromatography-mass spectrometry data. Nat Biotechnol. 2021;39(2):169–173. 10.1038/s41587-020-0700-3 PMC7971188 33169034

[136] Eva Gorrochategui JJ. Sílvia Lacorte, Romà Tauler, Data analysis strategies for targeted and untargeted LC-MS metabolomic studies: overview and workflow TrAC, Trends Anal Chem. 2016;82:425–442.

[137] Pinto J. Following healthy pregnancy by NMR metabolomics of plasma and correlation to urine. J Proteome Res. 2015;14(2);1263–1274. 10.1021/pr5011982 25529102

[138] Li X. Independent component analysis in non-hypothesis driven metabolomics: improvement of pattern discovery and simplification of biological data interpretation demonstrated with plasma samples of exercising humans. J Chromatogr B Analyt Technol Biomed Life Sci. 2012;910:156–162. 10.1016/j.jchromb.2012.06.030 22809791

[139] Monakhova YB. Independent components analysis to increase efficiency of discriminant analysis methods (FDA and LDA): application to NMR fingerprinting of wine. Talanta. 2015;141:60–65. 10.1016/j.talanta.2015.03.037 25966381

[140] Liu Y. MetICA: independent component analysis for high-resolution mass-spectrometry based non-targeted metabolomics. BMC Bioinformatics. 2016;17:114. 10.1186/s12859-016-0970-4 PMC4776428 26936354

[141] Ouyang M. Application of sparse linear discriminant analysis for metabolomics data. Anal Methods. 2014;6(22):9037–9044.

[142] Ledauphin J. Differences in the volatile compositions of French labeled brandies (Armagnac, Calvados, Cognac, and Mirabelle) using GC-MS and PLS-DA. J Agric Food Chem. 2010;58(13):7782–7793. 10.1021/jf9045667 20527953

[143] Safo SE, Li S, Long Q. Integrative analysis of transcriptomic and metabolomic data via sparse canonical correlation analysis with incorporation of biological information. Biometrics. 2018;74(1):300–312. 10.1111/biom.12715 PMC5677597 28482123

[144] Goodwin CR. Phenotypic mapping of metabolic profiles using self-organizing maps of high-dimensional mass spectrometry data. Anal Chem. 2014;86(13):6563–6571. 10.1021/ac5010794 PMC4082383 24856386

[145] Lin X. A method for handling metabonomics data from liquid chromatography/mass spectrometry: combinational use of support vector machine recursive feature elimination, genetic algorithm and random forest for feature selection. Metabolomics. 2011;7(4):549–558.

[146] Huang J-H. Distinguishing the serum metabolite profiles differences in breast cancer by gas chromatography mass spectrometry and random forest method. RSC Adv. 2015;5(73):58952–58958.

[147] Tebani A, Afonso C, Bekri S. Advances in metabolome information retrieval: turning chemistry into biology. Part II: biological information recovery. J Inherit Metab Dis. 2018;41(3):393–406. 10.1007/s10545-017-0080-0 PMC5959951 28842777

[148] Pinto RC. Chemometrics methods and strategies in metabolomics metabolomics: from fundamentals to clinical applications. Springer. 2017;163–190. 10.1007/978-3-319-47656-8_7 28132180

[149] Gorrochategui E. Data analysis strategies for targeted and untargeted LC-MS metabolomic studies: overview and workflow. TrAC Trends Anal Chem. 2016;82:425–442.

[150] Barr DB, Wang RY, Needham LL. Biologic monitoring of exposure to environmental chemicals throughout the life stages: requirements and issues for consideration for the National Children’s Study. Environ Health Perspect. 2005;113(8):1083–1091. 10.1289/ehp.7617 PMC1280353 16079083

[151] Seymour CW. Precision medicine for all? Challenges and opportunities for a precision medicine approach to critical illness. Crit Care. 2017;21(1):257. 10.1186/s13054-017-1836-5 PMC5648512 29047353

[152] Baum JLR, et al. Evaluation of silicone-based wristbands as passive sampling systems using PAHs as an exposure proxy for carcinogen monitoring in firefighters: evidence from the firefighter cancer initiative. Ecotoxicol Environ Saf. 2020;205:111100. 10.1016/j.ecoenv.2020.111100 32911453

[153] Caban-Martinez AJ. Objective measurement of carcinogens among Dominican Republic firefighters using silicone-based wristbands. J Occup Environ Med. 2020;62(11):e611–e615. 10.1097/JOM.0000000000002006 32826549

[154] Doherty BT, et al. Assessment of multipollutant exposures during pregnancy using silicone wristbands. Front Public Health. 2020;8:547239. 10.3389/fpubh.2020.547239 PMC7550746 33117768

[155] Travis SC, et al. Catching flame retardants and pesticides in silicone wristbands: evidence of exposure to current and legacy pollutants in Uruguayan children. Sci Total Environ. 2020;740:140136. 10.1016/j.scitotenv.2020.140136 PMC10989841 32927574

[156] Wang Y, et al. Measuring exposure of e-waste dismantlers in Dhaka Bangladesh to organophosphate esters and halogenated flame retardants using silicone wristbands and T-shirts. Sci Total Environ. 2020;720:137480. 10.1016/j.scitotenv.2020.137480 32146393

[157] Rohlman D, et al. A case study describing a community-engaged approach for evaluating polycyclic aromatic hydrocarbon exposure in a native American community. Int J Environ Res Public Health. 2019;16(3). 10.3390/ijerph16030327 PMC6388274 30682857

[158] Dixon HM, et al. Discovery of common chemical exposures across three continents using silicone wristbands. R Soc Open Sci. 2019;6(2):181836. 10.1098/rsos.181836 PMC6408398 30891293

[159] Gibson EA. Differential exposure to organophosphate flame retardants in mother-child pairs. Chemosphere. 2019;219:567–573. 10.1016/j.chemosphere.2018.12.008 PMC6460923 30553217

[160] Romanak KA. Analysis of brominated and chlorinated flame retardants, organophosphate esters, and polycyclic aromatic hydrocarbons in silicone wristbands used as personal passive samplers. J Chromatogr A. 2019;1588:41–47. 10.1016/j.chroma.2018.12.041 PMC6393176 30639062

[161] Paulik LB. Environmental and individual PAH exposures near rural natural gas extraction. Environ Pollut. 2018;241:397–405. 10.1016/j.envpol.2018.05.010 PMC7169985 29857308

